# Association between *tumor necrosis factor-alpha polymorphisms* (*rs361525*, *rs1800629*, *rs1799724*, *1800630*, and *rs1799964*) and risk of psoriasis in studies following Hardy-Weinberg equilibrium: A systematic review and meta-analysis

**DOI:** 10.1016/j.heliyon.2023.e17552

**Published:** 2023-06-22

**Authors:** Sepehr Sadafi, Ali Ebrahimi, Masoud Sadeghi, Omid Emami Aleagha

**Affiliations:** a Molecular Pathology Research Center, Imam Reza Hospital, Kermanshah University of Medical Sciences, Kermanshah, Iran; b Clinical Research Development Center, Imam Reza Hospital, Kermanshah University of Medical Sciences, Kermanshah, Iran; c Dermatology Department, Hajdaie Dermatology Clinic, Medicine School, Kermanshah University of Medical Sciences, Kermanshah, Iran; d Medical Biology Research Center, Kermanshah University of Medical Sciences, Kermanshah, Iran

**Keywords:** Psoriasis, Cytokine, Tumor necrosis factor-alpha, Polymorphism, meta-Analysis

## Abstract

**Objective:**

Psoriasis is a disease with an immunogenetic background in which cytokines have important effects on its prevalence and incidence. The present meta-analysis evaluated the relationship between *tumor necrosis factor-alpha* (*TNF-α*) polymorphisms (*rs361525*, *rs1800629*, *rs1799724*, *1800630*, and *rs1799964*) and psoriasis risk in studies following Hardy-Weinberg equilibrium (HWE).

**Materials and methods:**

Four databases were searched to retrieve relevant studies reporting the distributions of *TNF-α* polymorphisms in psoriasis cases compared to controls. The effect sizes were the 95% confidence intervals (CIs) and odds ratios (ORs). Subgroup analysis, sensitivity analyses, publication bias, trial sequential analysis (TSA), and meta-regression were performed on the initial pooled results of *TNF-α* polymorphisms.

**Results:**

Thirty-six articles with 71 studies were included in the meta-analysis (twenty-six: *rs*361525, twenty-seven: *rs*1800629, nine: *rs*1799724, four: *1800630*, and five: *rs*1799964). The pooled ORs for −238 G/A *rs*361525 polymorphism were 2.33 (*p* < 0.00001), 2.79 (*p* < 0.0001), 2.35 (*p* < 0.00001), 2.44 (*p* < 0.00001), and 2.45 (*p* < 0.00001), as well as 1.57 (*p* < 0.00001), 1.98 (*p* = 0.01), 1.61 (*p* < 0.00001), 1.64 (*p* < 0.00001), and 1.79 (*p* < 0.00001) for −857 C/T *rs*1799724 polymorphism in allelic, homozygous, heterozygous, dominant, and recessive models, respectively. Ethnicity, psoriasis type, and sample size affected the pooled results of *rs*361525, *rs*1800629, and *rs*1799724 polymorphisms. Based on TSA, there were just sufficient cases for −238 G/A *rs*361525 polymorphism in five genetic models and −857C/T *rs*1799724 polymorphism in allelic, heterozygous, and dominant models.

**Conclusions:**

The A allele and GA and GG genotypes of −238 G/A *rs*361525 polymorphism and T allele, TT and CT genotypes of −857C/T *rs*1799724 polymorphism were related to increased risks in psoriasis cases. Well-designed studies (with no deviation from HWE in controls) with more cases are recommended in the future.

## 1 Introduction

Psoriasis is a chronic inflammatory skin disease that affects the immune system and is accompanied by several co-morbidities, including atherosclerosis and cardiovascular diseases, psoriatic arthritis, and mental health conditions [[1], [2], [3]]. Psoriasis and its associated diseases can associate significant decreases in the quality of life and bear significant social costs [4].

The prevalence of psoriasis varies across the world [[5], [6], [7], [8]]. A recent meta-analysis reported that the incidence of psoriasis in adults varied from 30.3 in Taiwan to 321.0 per 100 000 person-years in Italy. In addition, psoriasis prevalence varied from 0.14% in East Asia to 1.99% in Australasia [9]. The psoriasis incidence identified a bimodal distribution pattern, with peaks in early-onset (35–44 years) and late-onset (65–74 years) psoriasis age classes. Late-onset psoriasis showed changes based on sex, and women were diagnosed earlier than men [9].

The interaction between multiple genetic and environmental risk factors can cause psoriasis as a common inflammatory skin disease [10,11] and this disease has an immunogenetic background [12]. The data obtained from meta-analyses [13,14] and genome-wide association studies (GWAS) [15,16] show the relationship between genetic variation and the prevalence of the disease.

Psoriasis is a T lymphocyte-mediated inflammatory disease [17] that Th1 cells show a dominant role in its initiation and maintenance [12]. Tumor necrosis factor-alpha (TNF-α) is a potent inflammatory cytokine that may have a significant role in psoriasis [18], and its gene is located in the highly polymorphic major histocompatibility complex region on chromosome 6p21.3 [19].

Several studies have investigated the relationship of single nucleotide polymorphism (SNP) of *rs*361525*, rs*1800629*, rs*1799724*, 1800630*, and *rs*1799964 in psoriasis cases compared to controls with different results [[20], [21], [22], [23], [24], [25], [26]]. Six meta-analyses [[27], [28], [29], [30], [31], [32]] reported *TNF-α* polymorphisms in psoriasis cases. One of them [27] searched databases until 2019 and reported *rs*361525*, rs1800629,* and *rs*1799724 polymorphisms, including that these polymorphisms could be associated with susceptibility to psoriasis in certain populations*.* Another meta-analysis [28] until 2012 reported *rs*361525*, rs*1800629*, rs*1799724*, 1800630,* and *rs*1799964 polymorphisms that the first three polymorphisms are associated with psoriasis susceptibility. The third meta-analysis [29], which included studies up until 2019, reported on *rs*1800629 polymorphism and conducted that this polymorphism was associated with psoriasis susceptibility. A fourth meta-analysis [30], which included studies up until 2012, reported on *rs*361525 and *rs*1800629 polymorphisms, with *rs*361525 polymorphism having an increased risk and *rs*1800629 polymorphism having a protective role in psoriasis cases, and this result was confirmed by fifth meta-analysis [31], which included studies up until 2012 confirmed this result*.* A sixth meta-analysis [32], which included studies up until 2006, reported on *rs*361525 and *rs*1800629 polymorphisms, including that these polymorphisms were associated with the risk of psoriasis.

Testing for Hardy-Weinberg equilibrium (HWE) is a crucial quality control step in identifying suitable polymorphisms for genetic studies [33]. A deviation from HWE in controls can introduce could bias the estimation of genetic effects, particularly in meta-analyses [[34], [35], [36]]. The previous meta-analyses have included studies with a deviation from HWE and varying sample size. To reduce bias, we deleted the studies with HWE deviations in their controls to reduce the bias, as well as those with small sample sizes [37] that can affect the reliability of the results [37,38]. The present meta-analysis focuses on the association between *TNF-α* polymorphisms (*rs361525*, *rs1800629*, *rs1799724*, *1800630*, and *rs1799964*) and susceptibility to psoriasis in studies that followed HWE and had suitable cases.

## 2 Materials and methods

### 2.1 Study design

The design of the meta-analysis was performed based on preferred reporting items for systematic reviews and meta-analyses (PRISMA) protocols. Also, the population-exposure-comparators-outcomes (PECO) question was: Are *TNF-α* polymorphisms associated with the risk of psoriasis? (human cases with and without psoriasis: P; psoriasis disease, E; psoriasis cases compared to controls: C; and prevalence of alleles and genotypes of *TNF-α* polymorphisms: O).

### 2.2 Study selection

The databases of Scopus, PubMed/Medline, Cochrane Library, and Web of Science until July 12, 2022, without any restrictions, were comprehensively searched. The key terms to search a database were: (“TNFα” or “TNF-a” or “TNF-α” or “TNF alpha” or “TNF-alpha” or “Tumor necrosis factor-alpha” or “Tumor necrosis factor-α” or “Tumor necrosis factor alpha”) and (“psoriasis” or “psoriatic”) and (“polymorphism*” or “allele” or “genotype*” or “variant*” or “SNP”). Moreover, the citations of the retrieved articles in relation to the subject were examined to make sure that no study was omitted and then the titles/abstracts of all articles meeting the criteria were assessed by one author (M.S.) and then downloaded. Another author (O.E.A.) re-tested the study selection process. Differences between the results of both authors were resolved by a short conversation.

### 2.3 Study criteria

Inclusion criteria: case-control or cohort studies including psoriasis cases and controls without receiving any treatment. 2) studies reporting the prevalence of alleles and genotypes of *TNF-α* polymorphisms (*rs361525*, *rs1800629*, *rs1799724*, *1800630*, and *rs1799964*) in psoriasis cases and controls. 3) studies included more than 100 individuals in psoriasis and control groups together. 4) studies following HWE for their controls. 5) Psoriasis cases including any subtype. 6) clinical subtypes of psoriasis (psoriasis vulgaris and psoriatic arthritis) were diagnosed based on the Moll and Wright or CASPAR criteria [39,40] or by clinical specialists. 7) psoriasis cases and controls had no systemic illnesses (e.g., autoimmune disorders, infection diseases, malignancies, etc.).

Exclusion criteria: case reports, meta-analyses, meeting abstracts, review articles, editorial articles, studies with insufficient data to calculate distributions of alleles and genotypes, studies without a control group, book chapters, and studies including psoriasis patients with other diseases were excluded.

### 2.4 Data extraction

Two authors (M.S. and S.S.) separately extracted the data of the articles imported in the analysis and another author (A.E.) resolved the disagreement between the authors.

### 2.5 Quality assessment

The quality assessment of the studies was performed by one author (M.S.) using the Newcastle-Ottawa scale (NOS) that number eight was considered the maximum score, and a score of ≥7 showed a high-quality study.

### 2.6 Statistical analyses

The effect sizes, including the 95% confidence interval (CI) and odds ratio (OR), of *TNF-α* polymorphisms (*rs361525*, *rs1800629*, *rs1799724*, *1800630*, and *rs1799964*) between psoriasis cases and controls were calculated using the Review Manager 5.3 (RevMan 5.3) software. A *p*-value (2-sided) less than 0.05 was considered statistically significant, and *P*_heterogeneity_ less than 0.10 (I^2^ > 50%) identified significant heterogeneity. Due to significant heterogeneity, a random-effects model [41] was utilize with the studies, if not, a fixed-effect model [42] was used. The subgroup analysis and meta-regression were also performed.

The sensitivity analyses, including “one-study-removed” and “cumulative” analyses, were conducted to evaluate the stability/consistency of pooled ORs. The publication bias degree was assessed using Egger’s test to assess the degree of asymmetry [43] and Begg’s test estimated the potential publication bias [44]. If the *p*-value (2-sided) of both tests were less than 0.10, it indicated the existence of the publication bias. Sensitivity analyses and publication bias were conducted using the Comprehensive Meta-Analysis version 2.0 (CMA 2.0) software.

To report false positive or false negative results from meta-analyses [45], trial sequential analysis (TSA) was accomplished using TSA software (version 0.9.5.10 beta), [46]. The required information size (RIS) was calculated with an α-risk of 5%, a β-risk of 20%, and a 2-sided border type. If the Z curve crossed the RIS line or followed the borderline or futility zone, enough cases were involved in the studies, and the conclusion was trustworthy or dependable. The analyses were conducted by one author (M.S.).

## 3 Results

### 3.1 Selection of studies

**Fig. 1:**
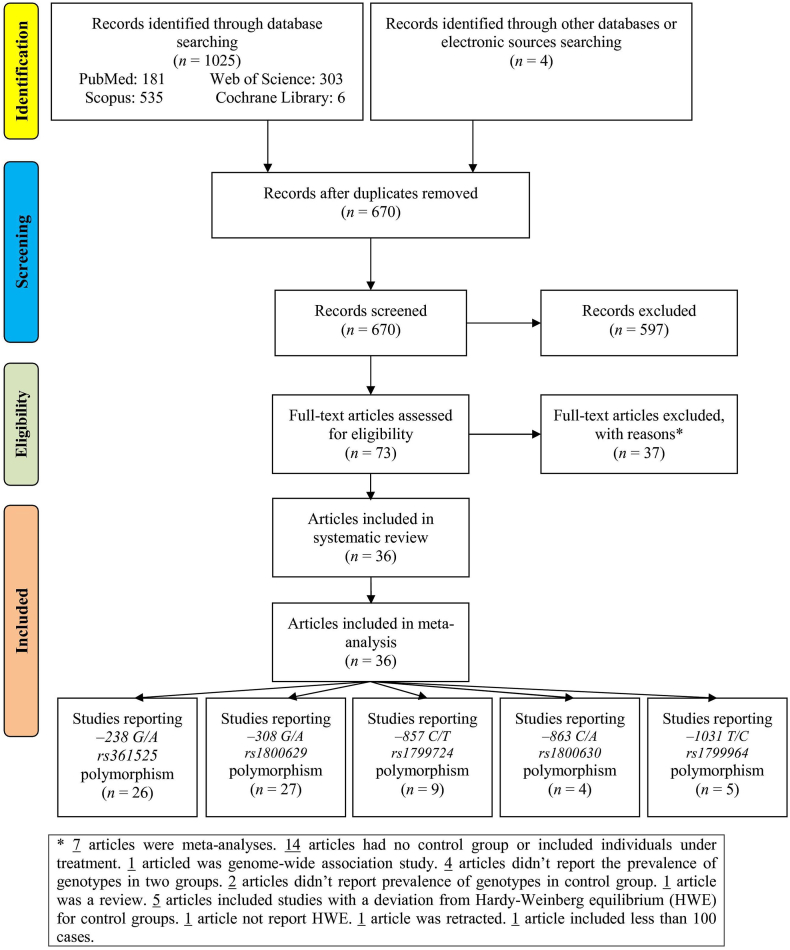
Flowchart of study selection.

Out of 1029 records recognized through databases and electronic sources, after eliminating duplicates, 670 remaining records were screened (Fig. 1). After that, 597 records were removed, 73 full-text articles were assessed for eligibility criteria. Of these, 37 articles were excluded for following reasons: seven meta-analyses, fourteen had no control group or included individuals under treatment, one genome-wide association study, four didn’t account for the prevalence of genotypes in two groups, two didn’t account the prevalence of genotypes in the control group, one review, five included studies with an HWE deviation for control groups, one didn’t report HWE, one retracted study, and one included less than 100 cases. At last, 36 articles were entered into the meta-analysis that they included 71 studies (26: *rs361525*, 27: *rs1800629*, 9: *rs1799724*, 4: *1800630*, and 5: *rs1799964*).

### 3.2 Features of studies

Table 1 illustrates the studies including −238 G/A *rs361525* polymorphism. Out of 24 articles [20,21,[23], [24], [25], [26],[47], [48], [49], [50], [51], [52], [53], [54], [55], [56], [57], [58], [59], [60], [61], [62], [63], [64]] that include 26 studies, fourteen studies were reported in Caucasians, eight in Asians, and four in mixed populations. Out of 26 studies, thirteen studies reported patients with psoriasis vulgaris, eight with psoriatic arthritis, four with psoriasis, and one with generalized pustular psoriasis.

**Table 1:** Characteristics of studies including −238 G/A *rs361525* polymorphism.

First author, publication year	Country	Ethnicity	Disease type	Sample size	Case			Control			P-value of HWE	Quality score
					GG	GA	AA	GG	GA	AA		
Höhler, 1997 (i) [47]	Germany	Caucasian	Psoriasis vulgaris	60/99	37	18	5	92	7	0	0.715	7
Höhler, 1997 (ii) [47]	Germany	Caucasian	Psoriatic arthritis	62/99	42	20	0	92	7	0	0.715	7
Reich, 1999 [49]	Germany	Caucasian	Psoriasis vulgaris	151/123	116	34	1	111	12	0	0.569	8
Jacob, 1999 [48]	Germany	Caucasian	Psoriasis vulgaris	83/99	57	26	0	92	7	0	0.715	6
Hamamoto, 2000 [25]	Japan	Asian	Psoriatic arthritis	20/87	20	0	0	83	4	0	0.826	7
González, 2001 [50]	Spain	Caucasian	Psoriatic arthritis	52/73	45	7	0	70	3	0	0.858	8
Al-Heresh, 2002 [51]	UK	Caucasian	Psoriatic arthritis	124/101	101	23	0	88	12	1	0.427	8
Reich, 2002 [52]	Germany	Caucasian	Psoriasis vulgaris	231/345	178	52	1	316	29	0	0.415	8
Zhang, 2002 [53]	China	Asian	Psoriasis vulgaris	87/128	68	19	0	114	12	2	0.575	7
Kim, 2003 [54]	Korea	Asian	Psoriasis vulgaris	103/125	89	14	0	111	13	1	0.383	8
Tsunemi, 2003 [55]	Japan	Asian	Psoriasis vulgaris	163/96	158	5	0	94	2	0	0.918	7
Long, 2004 [56]	China	Asian	Psoriasis vulgaris	77/82	64	10	3	79	3	0	0.866	8
Mössner, 2005 [57]	Germany	Caucasian	Psoriasis vulgaris	239/135	182	55	2	123	12	0	0.589	8
Rahman, 2006 [26]	Canada	Mixed	Psoriatic arthritis	427/204	345	80	2	183	19	2	0.075	7
Reich, 2007 (i) [23]	Germany	Caucasian	Psoriasis vulgaris	373/372	273	97	3	338	32	2	0.204	7
Reich, 2007 (ii) [23]	Germany	Caucasian	Psoriatic arthritis	368/372	303	60	5	338	32	2	0.204	7
Magalhães, 2010 [58]	Brazil	Mixed	Psoriasis	69/70	48	21	0	51	19	0	0.189	7
Popa, 2011 [24]	Romania	Caucasian	Psoriatic arthritis	86/147	78	8	0	141	6	0	0.801	8
Gallo, 2012 [20]	Spain	Caucasian	Psoriasis vulgaris	81/71	70	10	1	50	18	3	0.412	8
Wu, 2012 [59]	China	Asian	Psoriasis	107/110	80	23	4	99	10	1	0.213	8
Cardili, 2016 [22]	Brazil	Mixed	Psoriasis	125/202	91	34	0	162	40	0	0.118	8
Rajesh, 2017 [61]	India	Asian	Psoriasis vulgaris	72/72	35	34	3	64	8	0	0.618	7
Gulel, 2018 [21]	Turkey	Caucasian	Psoriasis	74/74	55	13	6	67	7	0	0.669	8
Herrera, 2018 [62]	Venezuela	Mixed	Psoriatic arthritis	52/52	48	4	0	41	11	0	0.393	8
Zhao, 2019 [63]	China	Asian	Generalized pustular psoriasis	91/102	77	12	2	98	4	0	0.839	8
Hagag, 2021 [64]	Egypt	Caucasian	Psoriasis vulgaris	70/70	35	24	11	48	19	3	0.531	8

HWE: Hardy-Weinberg equilibrium.

Table 2 illustrates the studies including −308 G/A *rs1800629* polymorphism. Twenty-two articles [20,[22], [23], [24],^26^,[49], [50], [51],[53], [54], [55], [56], [57], [58], [59],^62^,^63^,[65], [66], [67], [68], [69], [70], [71], [72], [73]] included 27 studies: fourteen were reported in Caucasians, nine in Asians, and four in mixed populations. Out of 27 studies, twelve reported patients with psoriasis vulgaris, six reported psoriatic arthritis, eight reported psoriasis, and one reported generalized pustular psoriasis diseases.

**Table 2:** Characteristics of studies including −308 G/A *rs1800629* polymorphism.

First author, publication year	Country	Ethnicity	Disease type	Sample size	Case			Control			P-value of HWE	Quality score
					GG	GA	AA	GG	GA	AA		
Reich, 1999 [49]	Germany	Caucasian	Psoriasis vulgaris	151/123	113	38	0	79	42	2	0.172	8
González, 2001 [50]	Spain	Caucasian	Psoriatic arthritis	52/73	44	8	0	58	15	0	0.328	8
Al-Heresh, 2002 [51]	UK	Caucasian	Psoriatic arthritis	124/101	79	38	7	71	27	3	0.825	8
Zhang, 2002 [53]	China	Asian	Psoriasis vulgaris	87/128	77	10	0	112	16	0	0.451	7
Kim, 2003 [54]	Korea	Asian	Psoriasis vulgaris	103/125	96	7	0	107	18	0	0.386	8
Tsunemi, 2003 [55]	Japan	Asian	Psoriasis vulgaris	163/96	161	2	0	92	4	0	0.835	7
Long, 2004 [56]	China	Asian	Psoriasis vulgaris	77/82	73	4	0	71	11	0	0.515	8
Mössner, 2005 [57]	Germany	Caucasian	Psoriasis vulgaris	239/135	197	41	1	93	39	1	0.151	8
Baran, 2006 [65]	Poland	Caucasian	Psoriasis vulgaris	78/74	66	12	0	57	16	1	0.918	7
Rahman, 2006 [26]	Canada	Mixed	Psoriatic arthritis	428/204	290	128	10	136	58	10	0.354	7
Nedoszytko, 2007 [66]	Poland	Caucasian	Psoriasis vulgaris	166/65	141	24	1	46	18	1	0.607	8
Reich, 2007(i) [23]	Germany	Caucasian	Psoriasis vulgaris	368/370	283	76	9	253	107	10	0.742	7
Reich, 2007 (ii) [23]	Germany	Caucasian	Psoriatic arthritis	361/370	275	76	10	253	107	10	0.742	7
Magalhães, 2010 [58]	Brazil	Mixed	Psoriasis	69/70	48	21	0	51	19	0	0.189	7
Popa, 2011 [24]	Romania	Caucasian	Psoriatic arthritis	86/142	71	13	2	107	30	5	0.130	8
Gallo, 2012 [20]	Spain	Caucasian	Psoriasis vulgaris	84/76	70	14	0	60	16	0	0.305	8
Zhou, 2012 [67]	China	Asian	Psoriasis	100/100	92	8	0	92	8	0	0.677	7
Wu, 2012 [59]	China	Asian	Psoriasis	107/110	97	9	1	104	6	0	0.769	8
Karam, 2014 [68]	Egypt	Caucasian	Psoriasis	110/129	39	53	18	28	60	32	0.421	7
Bergallo, 2015 [69]	Italy	Caucasian	Psoriasis	70/235	53	16	1	178	54	3	0.627	7
Moorchung, 2015 [70]	India	Asian	Psoriasis	112/243	7	99	6	163	71	9	0.715	8
Popadic, 2015 [71]	Serbia	Caucasian	Psoriasis vulgaris	130/259	106	23	1	196	62	1	0.090	8
Cardili, 2016 [22]	Brazil	Mixed	Psoriasis	125/202	88	34	3	170	29	3	0.187	7
Rajesh, 2017 [72]	India	Asian	Psoriasis vulgaris	74/74	64	9	1	61	13	0	0.407	7
Herrera, 2018 [62]	Venezuela	Mixed	Psoriatic arthritis	52/52	42	10	0	44	8	0	0.547	8
Zhao, 2019 [63]	China	Asian	Generalized pustular psoriasis	91/102	78	13	0	86	16	0	0.390	8
Hadi, 2020 [73]	Iraq	Caucasian	Psoriasis	100/100	33	49	18	53	44	3	0.083	7

HWE: Hardy-Weinberg equilibrium.

Table 3 illustrates the studies including −857 C/T, −863 C/A, and −1031 T/C polymorphisms. Eight articles [20,21,[23], [24], [25], [26],^60^,^63^] include nine studies for −857 C/T *rs1799724* polymorphism (6: Caucasians, 2: Asians and 1: mixed population). Out of nine studies, three reported patients with psoriasis vulgaris, four psoriatic arthritis, one just psoriasis without subtype, and one generalized pustular psoriasis. Three articles [25,26,74] included four studies reporting −863 C/A *rs1800630* polymorphism (two were reported in Caucasians, one in Asians, and one in a mixed population). Out of four studies, three included patients with psoriatic arthritis and one included psoriasis. Four articles [20,23,25,26] reporting −1031 T/C *rs1799964* polymorphism; included five studies that three studies were reported in Caucasians, one in Asians, and one in a mixed population. Out of five studies, two included cases with psoriasis vulgaris and three with psoriatic arthritis.

**Table 3:** Characteristics of studies including −857 C/T, −863C/A, and −1031 T/C polymorphisms.

First author, publication year	Country	Ethnicity	Disease type	Sample size	Case			Control			P-value of HWE	Quality score
*−*857 C/T *rs1799724*					CC	CT	TT	CC	CT	TT		
Hamamoto, 2000 [25]	Japan	Asian	Psoriatic arthritis	20/87	14	5	1	62	23	2	0.938	7
Rahman, 2006 [26]	Canada	Mixed	Psoriatic arthritis	419/204	339	71	9	174	27	3	0.249	7
Reich, 2007 (i) [23]	Germany	Caucasian	Psoriasis vulgaris	374/373	293	76	5	317	55	1	0.387	7
Reich, 2007 (ii) [23]	Germany	Caucasian	Psoriatic arthritis	370/373	275	92	3	317	55	1	0.387	7
Popa, 2011 [24]	Romania	Caucasian	Psoriatic arthritis	86/142	46	32	8	95	41	6	0.560	8
Gallo, 2012 [20]	Spain	Caucasian	Psoriasis vulgaris	77/76	58	18	1	65	10	1	0.406	8
Cabaleiro, 2013 [60]	Spain	Caucasian	Psoriasis vulgaris	142/160	102	38	2	135	23	2	0.378	7
Gulel, 2018 [21]	Turkey	Caucasian	Psoriasis	74/74	36	27	11	41	25	8	0.178	8
Zhao, 2019 [63]	China	Asian	Generalized pustular psoriasis	91/102	72	17	2	85	15	2	0.191	8
*−*863 C/A *rs1800630*					CC	CA	AA	CC	CA	AA		
Hamamoto, 2000 [25]	Japan	Asian	Psoriatic arthritis	20/87	15	4	1	63	21	3	0.487	7
Rahman, 2006 [26]	Canada	Mixed	Psoriatic arthritis	431/204	322	101	8	141	56	7	0.624	7
Smolnikova, 2019 (i) [74]	Russia	Caucasian	Psoriasis	77/103	65	12	0	79	24	0	0.180	8
Smolnikova, 2019 (ii) [74]	Russia	Caucasian	Psoriatic arthritis	99/103	77	19	3	79	24	0	0.180	8
*−*1031 T/C *rs1799964*					TT	TC	CC	TT	TC	CC		
Hamamoto, 2000 [25]	Japan	Asian	Psoriasis arthritis	20/87	16	4	0	65	22	0	0.177	7
Rahman, 2006 [26]	Canada	Mixed	Psoriatic arthritis	420/202	254	142	24	124	65	13	0.267	7
Reich, 2007 (i) [23]	Germany	Caucasian	Psoriasis vulgaris	370/368	204	146	20	212	138	18	0.458	7
Reich, 2007 (ii) [23]	Germany	Caucasian	Psoriatic arthritis	367/368	237	119	11	212	138	18	0.458	7
Gallo, 2012 [20]	Spain	Caucasian	Psoriasis vulgaris	86/72	55	28	3	33	30	9	0.595	8

HWE: Hardy-Weinberg equilibrium.

### 3.3 Meta-analysis

Forest plots of −238 G/A,−308 G/A*,*−857 C/T, −863 C/A, and −1031 T/C polymorphisms of *TNF-α* have been shown in Supplementary 1 and Table 4 shows the summary of pooled analysis results. The results reported that just −238 G/A and −857 C/T polymorphisms were associated with the risk of psoriasis. The pooled ORs for −238 G/A polymorphism were 2.33 (95%CI: 1.80, 3.02; *p*-value <0.00001), 2.79 (95%CI: 1.71, 4.55; *p*-value <0.0001), 2.35 (95%CI: 1.82, 3.04; *p*-value <0.00001), 2.44 (95%CI: 1.87, 3.19; *p*-value <0.00001), and 2.45 (95%CI: 1.50, 4.02; *p*-value <0.00001), as well as 1.57 (95%CI: 1.34, 1.84; *p*-value <0.00001), 1.98 (95%CI: 1.17, 3.35; *p*-value = 0.01), 1.61 (95%CI: 1.34, 1.93; *p*-value <0.00001), 1.64 (95%CI: 1.37, 1.95; *p*-value <0.00001), and 1.79 (95%CI: 1.07, 3.00; *p*-value <0.00001) for −857 C/T *rs1799724* polymorphism in allelic, homozygous, heterozygous, dominant, and recessive models*,* respectively. Therefore, A allele and GA and GG genotypes of −238 G/A polymorphism and T allele and TT and CT genotypes of −857 C/T polymorphism had elevated risks in psoriasis cases.

**Table 4:** Pooled analysis results of *–*238 G/A,*–*308 G/A*,–*857 C/T, *–*863C/A, and *–*1031 T/C polymorphisms.

Polymorphism (N)	Genetic model	OR	95%CI	*p*-value	Z	I^2^, %	*P*_heterogeneity_
*−*238 G/A *rs361525* [26]	A vs. G	2.33	1.80, 3.02	<0.00001	6.41	69	<0.00001
	AA vs. GG	2.79	1.71, 4.55	<0.0001	4.10	16	0.26
	GA vs. GG	2.35	1.82, 3.04	<0.00001	6.52	63	<0.00001
	AA + GA vs. GG	2.44	1.87, 3.19	<0.00001	6.54	67	<0.00001
	AA vs. GA + GG	2.45	1.50, 4.02	0.0004	3.57	6	0.38
*−*308 G/A *rs1800629* [27]	A vs. G	0.91	0.72, 1.16	0.47	0.73	81	<0.00001
	AA vs. GG	1.21	0.64, 2.29	0.55	0.60	64	0.0002
	GA vs. GG	0.88	0.66, 1.18	0.39	0.86	81	<0.00001
	AA + GA vs. GG	0.93	0.71, 1.23	0.62	0.50	80	<0.00001
	AA vs. GA + GG	1.01	0.75, 1.37	0.94	0.07	22	0.20
*−*857 C/T *rs1799724* [9]	T vs. C	1.57	1.34, 1.84	<0.00001	5.66	0	0.90
	TT vs. CC	1.98	1.17, 3.35	0.01	2.56	0	0.97
	CT vs. CC	1.61	1.34, 1.93	<0.00001	5.16	0	0.81
	TT + CT vs. CC	1.64	1.37, 1.95	<0.00001	5.50	0	0.83
	TT vs. CT + CC	1.79	1.07, 3.00	0.03	2.22	0	0.97
*−*863 C/A *rs1800630* [4]	A vs. C	0.77	0.60, 1.00	0.05	1.95	0	0.56
	AA vs. CC	0.88	0.37, 2.08	0.77	0.29	38	0.20
	CA vs. CC	0.76	0.57, 1.03	0.07	1.79	0	0.94
	AA + CA vs. CC	0.77	0.58, 1.03	0.08	1.77	0	0.85
	AA vs. CA + CC	0.93	0.39, 2.18	0.86	0.17	37	0.20
*−*1031 T/C *rs1799964* [5]	C vs. T	0.87	0.66, 1.15	0.34	0.96	67	0.02
	CC vs. TT	0.70	0.39, 1.25	0.22	1.22	51	0.11
	TC vs. TT	0.92	0.77, 1.10	0.34	0.95	27	0.24
	CC + TC vs. TT	0.89	0.65, 1.22	0.46	0.73	63	0.03
	CC vs. TC + TT	0.77	0.52, 1.12	0.17	1.37	32	0.22

### 3.4 Subgroup analyses

Subgroup analyses of −238 G/A *rs361525* polymorphism including ethnicity, psoriasis type, and sample size have been identified on five genetic models in Table 5. Ethnicity was an effective factor in the analyses of five genetic models, the sample size in the analysis of the recessive model, and psoriasis type in the analyses of homozygous and recessive models.

**Table 5:** Subgroup analysis results of −238 G/A *rs361525* polymorphism.

Polymorphism (N)	Genetic model	Subgroup (N)	OR	95%CI	*p*-value	Z	I^2^, %	*P*_heterogeneity_
*−*238 G/A *rs361525* [26]	A vs. G	Ethnicity						
		Caucasian [14]	2.67	1.91, 3.73	<0.00001	5.76	71	<0.0001
		Asian [8]	2.77	1.66, 4.63	0.0001	3.89	53	0.04
		Mixed [4]	1.22	0.75, 1.99	0.42	0.81	58	0.07
		Psoriasis type						
		Psoriasis vulgaris [13]	2.65	1.79, 3.94	<0.00001	4.84	76	<0.00001
		Psoriatic arthritis [8]	1.87	1.20, 2.91	0.006	2.78	55	0.03
		Sample size						
		≥200 [14]	2.28	1.95, 2.66	<0.00001	10.31	29	0.15
		<200 [12]	2.54	1.35, 4.77	0.004	2.90	83	<0.00001
	AA vs. GG	Ethnicity						
		Caucasian [14]	3.11	1.69, 5.73	0.0003	3.64	19	0.26
		Asian [8]	3.11	1.22, 7.96	0.02	2.37	5	0.39
		Mixed [4]	0.53	0.07, 3.80	0.53	0.63	–	–
		Psoriasis type						
		Psoriasis vulgaris [13]	2.85	1.55, 5.25	0.0007	3.37	21	0.25
		Psoriatic arthritis [8]	1.17	0.39, 3.46	0.78	0.28	17	0.30
		Sample size						
		≥200 [14]	1.74	0.90, 3.37	0.10	1.65	0	0.72
		<200 [12]	4.84	2.23, 10.50	<0.0001	3.99	45	0.10
	GA vs. GG	Ethnicity						
		Caucasian [14]	2.67	1.93, 3.70	<0.00001	5.90	64	0.0006
		Asian [8]	2.88	2.04, 4.07	<0.00001	6.00	36	0.14
		Mixed [4]	1.24	0.71, 2.16	0.45	0.75	61	0.05
		Psoriasis type						
		Psoriasis vulgaris [13]	2.75	1.87, 4.04	<0.00001	5.17	69	0.0001
		Psoriatic arthritis [8]	1.96	1.20, 3.19	0.007	2.71	58	0.02
		Sample size						
		≥200 [14]	2.45	2.07, 2.91	<0.00001	10.36	7	0.38
		<200 [12]	2.27	1.19, 4.31	0.01	2.50	79	<0.00001
	AA + GA vs. GG	Ethnicity						
		Caucasian [14]	2.79	1.99, 3.93	<0.00001	5.91	68	<0.0001
		Asian [8]	2.91	1.71, 4.94	<0.0001	3.94	51	0.05
		Mixed [4]	1.25	0.71, 2.21	0.44	0.77	64	0.04
		Psoriasis type						
		Psoriasis vulgaris [13]	2.85	1.90, 4.28	<0.00001	5.04	74	<0.00001
		Psoriatic arthritis [8]	1.96	1.21, 3.18	0.006	2.72	59	0.02
		Sample size						
		≥200 [14]	2.46	2.08, 2.90	<0.00001	10.60	16	0.28
		<200 [12]	2.50	1.28, 4.89	0.007	2.69	82	<0.00001
	AA vs. GA + GG	Ethnicity						
		Caucasian [14]	2.79	1.51, 5.14	0.001	3.28	8	0.37
		Asian [8]	2.60	1.01, 6.70	0.05	1.97	0	0.45
		Mixed [4]	0.48	0.07, 3.40	0.46	0.74	–	–
		Psoriasis type						
		Psoriasis vulgaris [13]	2.47	1.34, 4.56	0.004	2.89	6	0.39
		Psoriatic arthritis [8]	1.07	0.36, 3.17	0.91	0.12	18	0.30
		Sample size						
		≥200 [14]	1.53	0.79, 2.96	0.21	1.26	0	0.74
		<200 [12]	4.27	1.96, 9.32	0.0003	3.65	32	0.19

OR: Odds ratio. CI: Confidence interval.

Subgroup analyses of −308 G/A *rs1800629* polymorphism including ethnicity, psoriasis type, and sample size have been identified on five genetic models in Table 6. Ethnicity was an effective factor in the analysis of homozygous and dominant models; the sample size was another effective factor in allelic, homozygous, heterozygous, and dominant models, and psoriasis type in allelic, heterozygous, and dominant models.

**Table 6:** Subgroup analysis results of −308 G/A *rs1800629* polymorphism.

Polymorphism (N)	Genetic model	Subgroup (N)	OR	95%CI	*p*-value	Z	I^2^, %	*P*_heterogeneity_
*−*308 G/A *rs1800629* [27]	A vs. G	Ethnicity						
		Caucasian [14]	0.80	0.64, 0.99	0.04	2.07	64	0.0006
		Asian [9]	0.94	0.45, 1.94	0.87	0.17	86	<0.00001
		Mixed [4]	1.16	0.74, 1.81	0.51	0.65	64	0.04
		Psoriasis type						
		Psoriasis vulgaris [12]	0.65	0.55, 0.76	<0.00001	5.20	0	0.88
		Psoriatic arthritis [6]	0.86	0.72, 1.02	0.09	1.70	16	0.31
		Sample size						
		≥200 [19]	0.96	0.71, 1.30	0.81	0.25	86	<0.00001
		<200 [8]	0.79	0.61, 1.02	0.07	1.78	0	0.75
	AA vs. GG	Ethnicity						
		Caucasian [14]	0.94	0.49, 1.79	0.85	0.19	50	0.02
		Asian [9]	8.15	2.31, 28.72	0.001	3.26	0	0.43
		Mixed [4]	0.47	0.35, 1.56	0.42	0.80	34	0.22
		Psoriasis type						
		Psoriasis vulgaris [12]	0.69	0.34, 1.39	0.30	1.03	0	0.81
		Psoriatic arthritis [6]	0.80	0.47, 1.36	0.41	0.82	12	0.33
		Sample size						
		≥200 [19]	1.21	0.61, 2.42	0.58	0.55	70	<0.0001
		<200 [8]	1.14	0.23, 5.69	0.87	0.16	0	0.57
	GA vs. GG	Ethnicity						
		Caucasian [14]	0.68	0.54, 0.85	0.0009	3.33	54	0.008
		Asian [9]	1.06	0.38, 3.00	0.91	0.12	90	<0.00001
		Mixed [4]	1.22	0.76, 1.96	0.40	0.84	58	0.07
		Psoriasis type						
		Psoriasis vulgaris [12]	0.56	0.46, 0.67	<0.00001	6.15	0	0.51
		Psoriatic arthritis [6]	0.82	0.67, 1.01	0.06	1.87	45	0.11
		Sample size						
		≥200 [19]	0.96	0.64, 1.37	0.74	0.33	87	<0.00001
		<200 [8]	0.75	0.57, 0.99	0.05	2.00	0	0.73
	AA + GA vs. GG	Ethnicity						
		Caucasian [14]	0.75	0.61, 0.93	0.009	2.60	52	0.01
		Asian [9]	1.08	0.39, 3.01	0.88	0.15	90	<0.00001
		Mixed [4]	1.20	0.75, 1.94	0.45	0.76	62	0.05
		Psoriasis type						
		Psoriasis vulgaris [12]	0.60	0.50, 0.72	<0.00001	5.50	0	0.91
		Psoriatic arthritis [6]	0.84	0.69, 1.03	0.09	1.68	21	0.28
		Sample size						
		≥200 [19]	1.01	0.71, 1.44	0.95	0.06	86	<0.00001
		<200 [8]	0.76	0.57, 1.00	0.05	1.95	0	0.73
	AA vs. GA + GG	Ethnicity						
		Caucasian [14]	1.03	0.73, 1.46	0.86	0.18	31	0.14
		Asian [9]	1.72	0.67, 4.40	0.26	1.13	0	0.85
		Mixed [4]	0.71	0.33, 1.50	0.36	0.91	25	0.26
		Psoriasis type						
		Psoriasis vulgaris [12]	0.77	0.38, 1.56	0.47	0.72	0	0.82
		Psoriatic arthritis [6]	0.83	0.49, 1.41	0.48	0.70	12	0.33
		Sample size						
		≥200 [19]	1.00	0.74, 1.36	0.98	0.02	33	0.11
		<200 [8]	1.23	0.25, 6.11	0.80	0.25	0	0.57

OR: Odds ratio. CI: Confidence interval.

Subgroup analyses of −857C/T *rs1799724* polymorphism including ethnicity, psoriasis type, and sample size have been identified on five genetic models in Table 7. Ethnicity was an effective factor in the analysis of allelic, homozygous, heterozygous, and recessive models, psoriasis type in the homozygous model, and sample size in allelic, homozygous, heterozygous, and dominant models. Due to a low number of studies, we didn’t use the subgroup analysis for − 863 C/A *rs1800630* and −1031 T/C *rs1799964* polymorphisms.

**Table 7:** Subgroup analysis results of −857C/T *rs1799724* polymorphism.

Polymorphism (N)	Genetic model	Subgroup (N)	OR	95%CI	*p*-value	Z	I^2^, %	*P*_heterogeneity_
*−*857 C/T *rs1799724* [9]	T vs. C	Ethnicity						
		Caucasian [6]	1.67	1.39, 1.99	<0.00001	5.59	0	0.89
		Asian [2]	1.23	0.72, 2.09	0.44	0.76	0	0.87
		Psoriasis type						
		Psoriasis vulgaris [3]	1.67	1.28, 2.19	0.0002	3.73	0	0.84
		Psoriatic arthritis [4]	1.61	1.29, 2.01	<0.0001	4.20	0	0.61
		Sample size						
		≥200 [6]	1.62	1.36, 1.93	<0.00001	5.46	0	0.82
		<200 [3]	1.37	0.94, 1.99	0.10	1.64	0	0.75
	TT vs. CC	Ethnicity						
		Caucasian [6]	2.19	1.19, 4.05	0.01	2.51	0	0.86
		Asian [2]	1.47	0.31, 7.05	0.63	0.49	0	0.70
		Psoriasis type						
		Psoriasis vulgaris [3]	2.36	0.70, 7.97	0.17	1.38	0	0.56
		Psoriatic arthritis [4]	2.26	1.05, 4.88	0.04	2.09	0	0.90
		Sample size						
		≥200 [6]	2.23	1.16, 4.27	0.02	2.40	0	0.86
		<200 [3]	1.57	0.32, 3.84	0.32	0.99	0	0.94
	CT vs. CC	Ethnicity						
		Caucasian [6]	1.72	1.40, 2.11	<0.00001	5.15	0	0.76
		Asian [2]	1.21	0.64, 2.26	0.56	0.59	0	0.64
		Psoriasis type						
		Psoriasis vulgaris [3]	1.72	1.28, 2.31	0.0004	3.56	0	0.52
		Psoriatic arthritis [4]	1.62	1.26, 2.09	0.0002	3.73	0	0.53
		Sample size						
		≥200 [6]	1.65	1.36, 2.01	<0.00001	5.01	0	0.73
		<200 [3]	1.39	0.86, 2.25	0.18	1.33	0	0.53
	TT + CT vs. CC	Ethnicity						
		Caucasian [6]	1.75	1.43, 2.14	<0.00001	5.47	0	0.81
		Asian [2]	1.68	1.22, 2.33	0.002	3.13	0	0.61
		Psoriasis type						
		Psoriasis vulgaris [3]	1.74	1.30, 2.34	0.0002	3.74	0	0.66
		Psoriatic arthritis [4]	1.67	1.30, 2.13	<0.0001	4.05	0	0.54
		Sample size						
		≥200 [6]	1.68	1.39, 2.04	<0.00001	5.36	0	0.75
		<200 [3]	1.39	0.88, 2.19	0.16	1.42	0	0.61
	TT vs. CT + CC	Ethnicity						
		Caucasian [6]	1.93	1.06, 3.52	0.03	2.16	0	0.87
		Asian [2]	1.43	0.30, 6.83	0.65	0.45	0	0.67
		Psoriasis type						
		Psoriasis vulgaris [3]	2.10	0.63, 7.04	0.23	1.20	0	0.52
		Psoriatic arthritis [4]	2.05	0.96, 4.37	0.06	1.85	0	0.94
		Sample size						
		≥200 [6]	2.00	1.05, 3.82	0.03	2.11	0	0.88
		<200 [3]	1.45	0.61, 3.45	0.40	0.85	0	0.91

OR: Odds ratio. CI: Confidence interval.

### 3.5 Meta-regression

Meta-regression analyses (publication bias and sample size) of −238 G/A *rs361525*,−308 G/A *rs1800629,*−857 C/T *rs1799724* polymorphisms of *TNF-α* have been shown in Table 8. Publication year was a confounding factor on allelic, heterozygous, dominant models of −238 G/A *rs361525* polymorphism; as well as on five models of −308 G/A *rs1800629,* and also sample size was another confounding factor on allelic and dominant models of −308 G/A *rs1800629* polymorphism*.* Due to a low number of studies, we didn’t calculate the meta-regression analysis for −863 C/A and −1031 T/C polymorphisms.

**Table 8:** Subgroup analysis results of three polymorphisms.

Polymorphism (N)	Genetic model	Variable	Point estimate	Standard error	Lower limit	Upper limit	Z-value	*p*-value
*−*238 G/A *rs361525* [26]	A vs. G	Publication year	˗ 0.02323	0.01047	˗ 0.04375	˗ 0.00271	˗ 2.21882	0.02650
		Sample size	0.00027	0.00028	˗ 0.00028	0.00083	0.95708	0.33853
	AA vs. GG	Publication year	0.05264	0.04004	˗ 0.02583	0.13111	1.31483	0.18857
		Sample size	˗ 0.00119	0.00116	˗ 0.00347	0.00109	˗ 1.02329	0.30617
	GA vs. GG	Publication year	˗ 0.03611	0.01176	˗ 0.05915	˗ 0.01306	˗ 3.07122	0.00213
		Sample size	0.00044	0.00031	˗ 0.00018	0.00105	1.39930	0.16172
	AA + GA vs. GG	Publication year	˗ 0.02971	0.01147	˗ 0.05219	˗ 0.00723	˗ 2.59001	0.00960
		Sample size	0.00035	0.00030	˗ 0.00025	0.00094	1.13509	0.25634
	AA vs. GA + GG	Publication year	0.05097	0.03969	˗ 0.02683	0.12877	1.28404	0.19913
		Sample size	˗ 0.00118	0.00116	˗ 0.00345	0.00109	˗ 1.01666	0.30932
*−*308 G/A *rs1800629* [27]	A vs. G	Publication year	0.05919	0.00904	0.04147	0.07691	6.54594	<0.00001
		Sample size	˗ 0.00056	0.00023	˗ 0.00101	˗ 0.00012	˗ 2.46782	0.01359
	AA vs. GG	Publication year	0.10580	0.03414	0.03888	0.17272	3.09863	0.00194
		Sample size	˗ 0.00103	0.00076	˗ 0.00253	0.00046	˗ 1.35185	0.17642
	GA vs. GG	Publication year	0.06138	0.01124	0.03935	0.08342	5.45995	<0.00001
		Sample size	˗ 0.00034	0.00027	˗ 0.00087	0.00018	˗ 1.28011	0.20051
	AA + GA vs. GG	Publication year	0.05511	0.01081	0.03393	0.07630	5.09857	<0.00001
		Sample size	˗ 0.00051	0.00026	˗ 0.00102	˗ <0.00001	˗ 1.96763	0.04911
	AA vs. GA + GG	Publication year	0.06843	0.03277	0.00420	0.13267	2.08800	0.03680
		Sample size	˗ 0.00068	0.00074	˗ 0.00213	0.00077	˗ 0.92302	0.35599
*−*857 C/T *rs1799724* [9]	T vs. C	Publication year	˗ 0.00577	0.01749	˗ 0.04004	0.02850	˗ 0.33001	0.74139
		Sample size	0.00019	0.00031	˗ 0.00042	0.00079	0.60472	0.54536
	TT vs. CC	Publication year	˗ 0.03537	0.05028	˗ 0.13392	0.06319	˗ 0.70333	0.48185
		Sample size	0.00077	0.00121	˗ 0.00160	0.00314	0.63602	0.52476
	CT vs. CC	Publication year	0.00155	0.02148	˗ 0.04054	0.04365	0.07235	0.94232
		Sample size	0.00013	0.00037	˗ 0.00058	0.00085	0.36557	0.71468
	TT + CT vs. CC	Publication year	˗ 0.00250	0.02035	˗ 0.04238	0.03738	˗ 0.12279	0.90227
		Sample size	0.00016	0.00035	˗ 0.00053	0.00085	0.45723	0.64750
	TT vs. CT + CC	Publication year	˗ 0.03700	0.04944	˗ 0.13390	0.05990	˗ 0.74836	0.45424
		Sample size	0.00081	0.00120	˗ 0.00154	0.00316	0.67450	0.49999

### 3.6 Sensitivity analysis

The plots of sensitivity analyses for −238 G/A*,*−308 G/A*,* and −857 C/T polymorphisms of *TNF-α* including “one-study-removed” and “cumulative analysis” have been shown in Supplementary 2 that the results showed the stability of the pooled analyses. Due to a low number of studies, we didn’t calculate the sensitivity analysis for −863 C/A and −1031 T/C polymorphisms.

### 3.7 Trial sequential analysis (TSA)

**Fig. 2:**
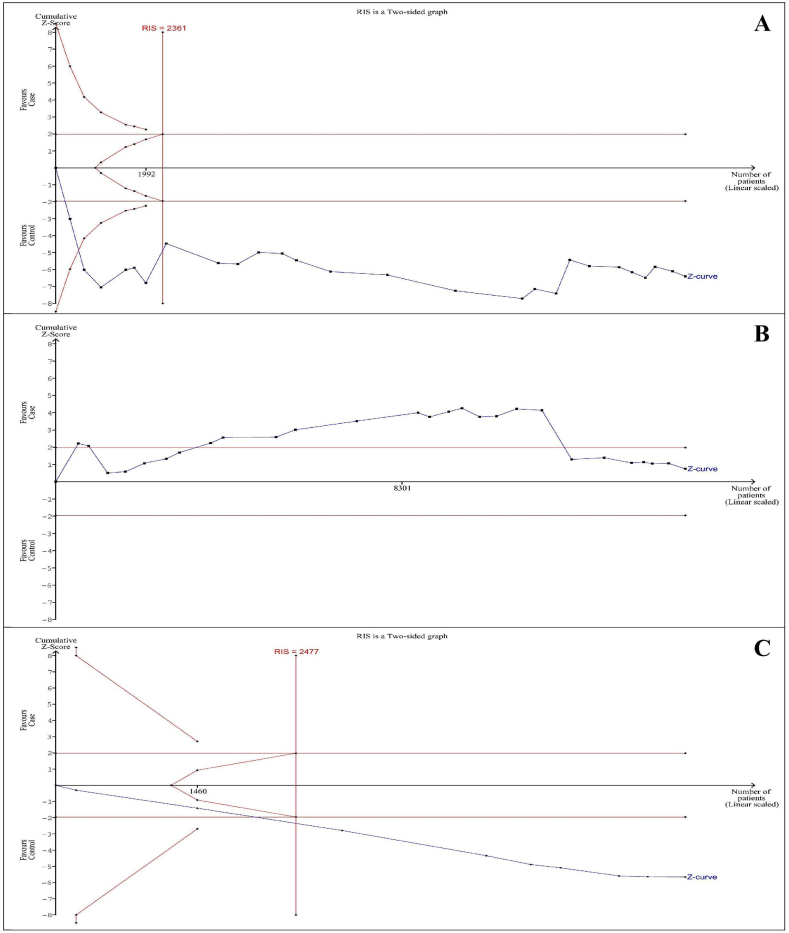
Trial sequential analysis of allelic model. A) −238 G/A *rs361525* polymorphism (D^2^ = 73%). B) −308 G/A *rs1800629* polymorphism (D^2^ = 84%). C) −857C/T *rs1799724* polymorphism (D^2^ = 0%).

Fig. 2 shows the TSA of the allelic model for −238 G/A*,*−308 G/A*,* and −857 C/T polymorphisms of *TNF-α*, and also Supplementary 4 shows the TSA of other models for three polymorphisms. Due to a low number of studies, we didn’t calculate the TSA for −863 C/A *rs1800630* and −1031 T/C *rs1799964* polymorphisms. The results showed that Z-curve crossed the RIS line for −238 G/A *rs361525* polymorphism, but not for −308 G/A *rs1800629* polymorphism in five genetic models. With regards to −857 C/T *rs1799724* polymorphism, the RIS line was just reached by Z-curve in allelic, heterozygous, and dominant models. Therefore, there were just sufficient cases for −238 G/A *rs361525* polymorphism in five genetic models and −857C/T *rs1799724* polymorphism in allelic, heterozygous, and dominant models.

### 3.8 Publication bias

Funnel plots of −238 G/A*,*−308 G/A*,* and −857 C/T polymorphisms of *TNF-α* have been shown in Supplementary 3. The *p*-values of Egger’s/Begg’s tests were 0.855/0.707, 0.970/0.216, 0.652/0.842, 0.840/0.912, and 0.935/0.248 for −238 G/A *rs361525* polymorphism; 0.474/0.631, 0.545/0.804, 0.573/0.545, 0.491/0.491, and 0.510/0.869 for −308 G/A *rs1800629* polymorphism; and 0.272/0.404, 0.823/0.834, 0.364/0.297, 0.324/0.404, and 0.755/0.676 for −857 C/T *rs1799724* polymorphism in allelic, homozygous, heterozygous, dominant, and recessive models, respectively. There was no publication bias for the pooled results of three polymorphisms. The due to low numbers of studies, we didn’t calculate the publication bias for −863 C/A and −1031 T/C polymorphisms.

## 4 Discussion

The main results of the meta-analysis recommended that A allele and GA and GG genotypes of −238 G/A *rs361525* polymorphism, as well as T allele and TT and CT genotypes of −857 C/T *rs1799724* polymorphism were associated with elevated risks in psoriasis cases. There was no association between −308 G/A*,* −863 C/A, and −1031 T/C polymorphisms and the risk of psoriasis. Ethnicity, psoriasis type, and sample size were effective factors in the pooled results of −238 G/A*,* −308 G/A*, and* −857 C/T polymorphisms*.* The number of cases was not sufficient for the pooled analysis of −308 G/A polymorphism.

Psoriasis is in a state of oxidative stress [75] and its pathogenesis is complex [76]. Cytokines play a significant role in mediating cell-cell interactions that lead to abnormal structures and functions of plenty of cell types in this disease [76]. The meta-analyses [[27], [28], [29], [30], [31], [32]] reported the *TNF-α* polymorphisms were associated with psoriasis risk. The meta-analysis of Zhu et al. [28] reported that −308A/G polymorphism is in relation to psoriasis susceptibility and five other meta-analyses [[29], [30], [31], [32]] confirmed this result, whereas the present meta-analysis rejected the relationship between −308A/G polymorphism and the risk of psoriasis.

All meta-analyses reporting −238 G/A *rs361525* polymorphism [27,28,[30], [31], [32]] recommended that this polymorphism was in relation to the risk of psoriasis and the result was in accordance with the result of the present meta-analysis. One of them [27], reported that −857 C/T *rs1799724* polymorphism was significantly related to psoriasis in Caucasians and the present meta-analysis confirmed it, but the result was in contrast with the result of the meta-analysis of Zhu and his colleagues [28]. Zhu et al. [28] in their meta-analysis reported that −857 C/T *rs1799724* polymorphism was related to psoriatic arthritis risk that the present meta-analysis identified that this polymorphism was associated with both psoriasis vulgaris and psoriatic arthritis. In addition, the polymorphisms of −308 G/A, −863 C/A, and −1031 T/C were not related to the psoriasis susceptibility, as well as the present meta-analysis confirmed it.

The accurate mechanism of ethnic disparity is unclear, but studying variations in genetic base and social agents across different people may be important [29]. Ethnically different populations can have different cultures and lifestyles that may present various genetics and the risk of certain diseases [29]. As this meta-analysis reported the association between −238 G/A and −308 G/A polymorphism and the psoriasis risk related to ethnicity.

The relationship between types of polymorphisms and the risk of psoriasis is related to psoriasis type [77,78] including a meta-analysis [28] reported the different associations between *TNF-α* polymorphisms in two types of psoriasis (psoriasis vulgaris and psoriatic arthritis) that these results can suggest stronger heritability in a number of psoriasis types [77]. As well as that the present meta-analysis showed that the association between −238 G/A,−308 G/A*,* and −857 C/T polymorphisms and psoriasis risk can be related to psoriasis type.

In this study, publication year, HWE, ethnicity, psoriasis type, and sample size could be probable confounding factors in the results. One way to reduce these effects would be the researchers to conduct additional studies including the controls without a deviation of HWE across diverse ethnic groups, each study focusing on a specific subtype of psoriasis.

It is important to note that the meta-analysis had two important limitations: firstly, only published studies were included in the analysis; secondly, there was low representation of certain polymorphisms in terms of the number of studies and individuals. Nonetheless, the strengths of the meta-analysis include high-quality scores in the studies, lack of publication bias, and the stability of results for −238 G/A,−308 G/A*,* and −857 C/T polymorphisms.

## 5 Conclusions

The meta-analysis recommended that the A allele and GA and GG genotypes of −238 G/A polymorphism, as well as the T allele and TT and CT genotypes of −857 C/T polymorphism were related to increased risks in psoriasis cases. There was no relationship between −308 G/A*,* −863 C/A*,* and −1031 T/C polymorphisms and the susceptibility to psoriasis. The −238 G/A,−308 G/A*,* and −857 C/T polymorphisms are playing the roles in psoriasis risk in an ethnicity-, psoriasis type, and sample size-related manner. A number of polymorphisms included low studies and individuals and therefore, well-designed studies (with no deviation from HWE in controls) with more are recommended in the future.

## Author contribution statement

Sepehr Sadafi: Conceived and designed the experiments; Contributed reagents, materials, analysis tools or data.

Ali Ebrahimi: Contributed reagents, materials, analysis tools or data.

Masoud Sadeghi: Performed the experiments; Analyzed and interpreted the data; Contributed reagents, materials, analysis tools or data.

Omid Emami Aleagha: Contributed reagents, materials, analysis tools or data; Wrote the paper.

## Data availability statement

Data included in article/supp. material/referenced in article.

## Declaration of competing interest

The authors declare that they have no known competing financial interests or personal relationships that could have appeared to influence the work reported in this paper
