# Supplementary material for: Association between tumor necrosis factor-alpha polymorphisms (rs361525, rs1800629, rs1799724, 1800630, and rs1799964) and risk of psoriasis in studies following Hardy-Weinberg equilibrium: A systematic review and meta-analysis
Source: Heliyon. 2023 Jun 22;9(7):e17552. doi: 10.1016/j.heliyon.2023.e17552 (PMC10338315; doi:10.1016/j.heliyon.2023.e17552)
Supplement: Multimedia component 2 [file mmc2.docx]

**Figure 1S**: One-study-removed analysis of association between *–238 G/A rs361525* polymorphism and the risk of psoriasis based on allelic model (A vs. G)

**Figure 2S**: One-study-removed analysis of association between *–238 G/A rs361525* polymorphism and the risk of psoriasis based on homozygous model (AA vs. GG)

**Figure 3S**: One-study-removed analysis of association between *–238 G/A rs361525* polymorphism and the risk of psoriasis based on heterozygous model (GA vs. GG)

**Figure 4S**: One-study-removed analysis of association between *–238 G/A rs361525* polymorphism and the risk of psoriasis based on dominant model (AA + GA vs. GG)

**Figure 5S**: One-study-removed analysis of association between *–238 G/A rs361525* polymorphism and the risk of psoriasis based on recessive model (AA vs. GA + GG)

**Figure 6S**: Cumulative analysis of association between *–238 G/A rs361525 polymorphism* and the risk of psoriasis based on allelic model (A vs. G)

**Figure 7S**: Cumulative analysis of association between *–238 G/A rs361525 polymorphism* and the risk of psoriasis based on homozygous model (AA vs. GG)

**Figure 8S**: Cumulative analysis of association between *–238 G/A rs361525 polymorphism* and the risk of psoriasis based on heterozygous model (GA vs. GG)

**Figure 9S**: Cumulative analysis of association between *–238 G/A rs361525 polymorphism* and the risk of psoriasis based on dominant model (AA + GA vs. GG)

**Figure 10S**: Cumulative analysis of association between *–238 G/A rs361525 polymorphism* and the risk of psoriasis based on recessive model (AA vs. GA + GG)

**Figure 11S**: One-study-removed analysis of association between *–308 G/A rs1800629* *polymorphism* and the risk of psoriasis based on allelic model (A vs. G)

**Figure 12S**: One-study-removed analysis of association between *–308 G/A rs1800629* *polymorphism* and the risk of psoriasis based on homozygous model (AA vs. GG)

**Figure 13S**: One-study-removed analysis of association between *–308 G/A rs1800629* *polymorphism* and the risk of psoriasis based on heterozygous model (GA vs. GG)

**Figure 14S**: One-study-removed analysis of association between *–308 G/A rs1800629* *polymorphism* and the risk of psoriasis based on dominant model (AA + GA vs. GG)

**Figure 15S**: One-study-removed analysis of association between *–308 G/A rs1800629* *polymorphism* and the risk of psoriasis based on recessive model (AA vs. GA + GG)

**Figure 16S**: Cumulative analysis of association between *–308 G/A rs1800629* *polymorphism* and the risk of psoriasis based on allelic model (A vs. G)

**Figure 17S**: Cumulative analysis of association between *–308 G/A rs1800629* *polymorphism* and the risk of psoriasis based on homozygous model (AA vs. GG)

**Figure 18S**: Cumulative analysis of association between *–308 G/A rs1800629* *polymorphism* and the risk of psoriasis based on heterozygous model (GA vs. GG)

**Figure 19S**: Cumulative analysis of association between *–308 G/A rs1800629* *polymorphism* and the risk of psoriasis based on dominant model (AA + GA vs. GG)

**Figure 20S**: Cumulative analysis of association between *–308 G/A rs1800629* *polymorphism* and the risk of psoriasis based on recessive model (AA vs. GA + GG)

**Figure 21S**: One-study-removed analysis of association between *–857 C/T rs1799724 polymorphism* and the risk of psoriasis based on allelic model (T vs. C)

**Figure 22S**: One-study-removed analysis of association between *–857 C/T rs1799724 polymorphism* and the risk of psoriasis based on homozygous model (TT vs. CC)

**Figure 23S**: Fun One-study-removed analysis of association between *–857 C/T rs1799724 polymorphism* and the risk of psoriasis based on heterozygous model (CT vs. CC)

**Figure 24S**: One-study-removed analysis of association between *–857 C/T rs1799724 polymorphism* and the risk of psoriasis based on dominant model (TT + CT vs. CC)

**Figure 25S**: One-study-removed analysis of association between *–857 C/T rs1799724 polymorphism* and the risk of psoriasis based on recessive model (TT vs. CT + CC)

**Figure 26S**: Cumulative analysis of association between *–857 C/T rs1799724 polymorphism* and the risk of psoriasis based on allelic model (T vs. C)

**Figure 27S**: Cumulative analysis of association between *–857 C/T rs1799724 polymorphism* and the risk of psoriasis based on homozygous model (TT vs. CC)

**Figure 28S**: Cumulative analysis of association between *–857 C/T rs1799724 polymorphism* and the risk of psoriasis based on heterozygous model (CT vs. CC)

**Figure 29S**: Cumulative analysis of association between *–857 C/T rs1799724 polymorphism* and the risk of psoriasis based on dominant model (TT + CT vs. CC)

**Figure 30S**: Cumulative analysis of association between *–857 C/T rs1799724 polymorphism* and the risk of psoriasis based on recessive model (TT vs. CT + CC)
