# Supplementary material for: Association between tumor necrosis factor-alpha polymorphisms (rs361525, rs1800629, rs1799724, 1800630, and rs1799964) and risk of psoriasis in studies following Hardy-Weinberg equilibrium: A systematic review and meta-analysis
Source: Heliyon. 2023 Jun 22;9(7):e17552. doi: 10.1016/j.heliyon.2023.e17552 (PMC10338315; doi:10.1016/j.heliyon.2023.e17552)
Supplement: Multimedia component 3 [file mmc3.docx]

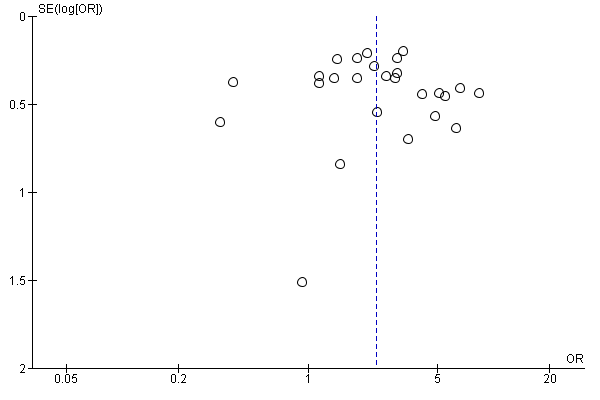


**Figure 1S**: Funnel plot analysis of association between *–238 G/A rs361525 polymorphism* and the risk of psoriasis based on allelic model (A vs. G)


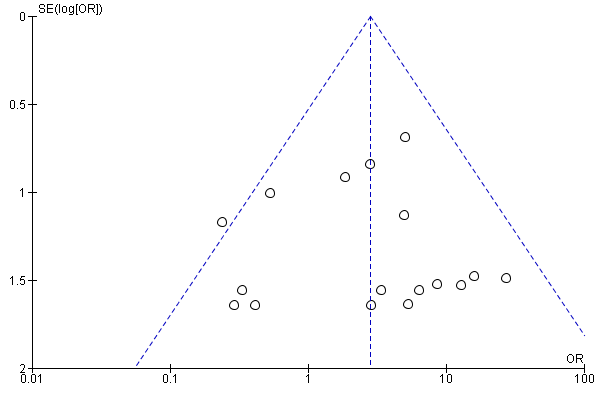


**Figure 2S**: Funnel plot analysis of association between *–238 G/A rs361525 polymorphism* and the risk of psoriasis based on homozygous model (AA vs. GG)


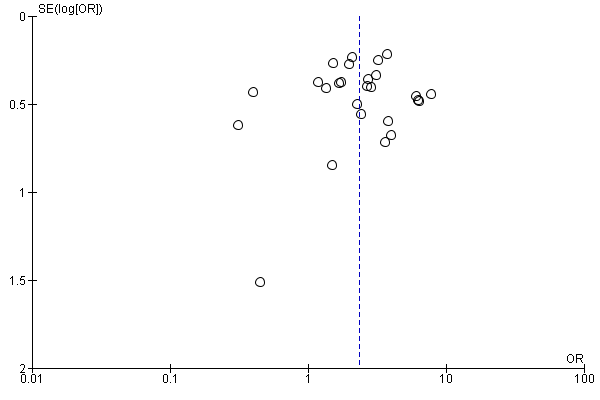


**Figure 3S**: Funnel plot analysis of association between *–238 G/A rs361525 polymorphism* and the risk of psoriasis based on heterozygous model (GA vs. GG)


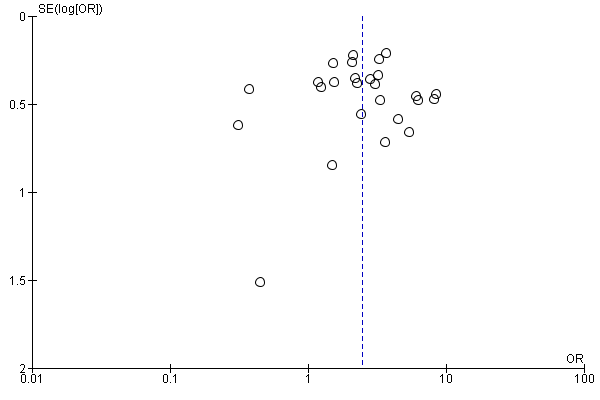


**Figure 4S**: Funnel plot analysis of association between *–238 G/A rs361525 polymorphism* and the risk of psoriasis based on dominant model (AA + GA vs. GG)


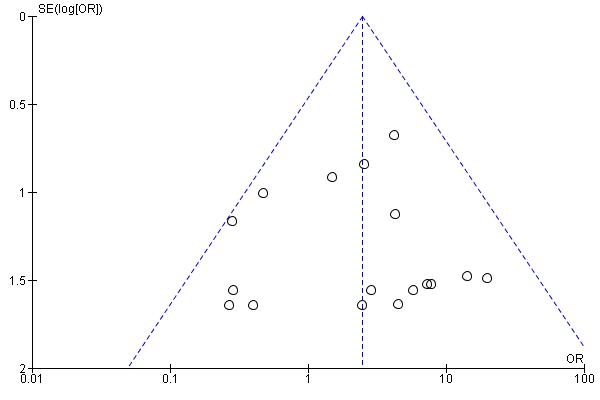


**Figure 5S**: Funnel plot analysis of association between *–238 G/A rs361525 polymorphism* and the risk of psoriasis based on recessive model (AA vs. GA + GG)

**
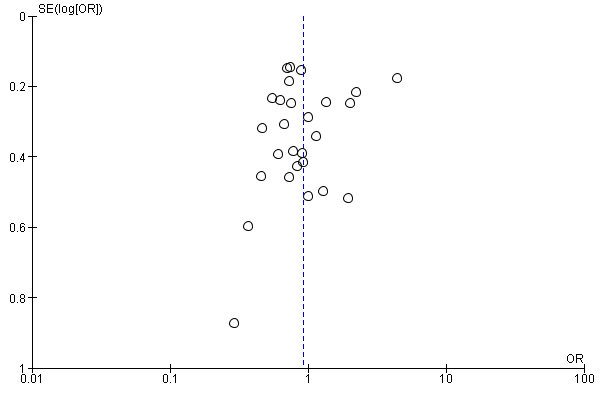
**

**Figure 6S**: Funnel plot analysis of association between *–308 G/A rs1800629* *polymorphism* and the risk of psoriasis based on allelic model (A vs. G)


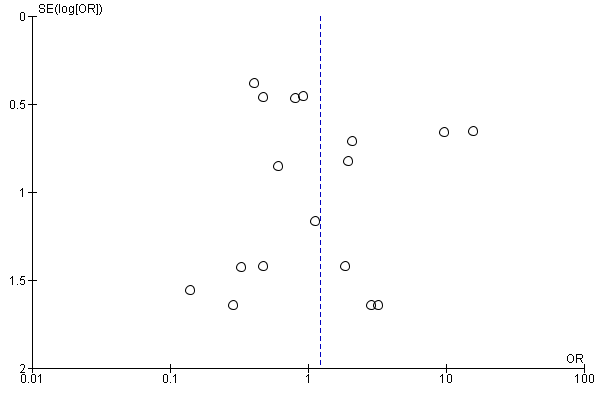


**Figure 7S**: Funnel plot analysis of association between *–308 G/A rs1800629* *polymorphism* and the risk of psoriasis based on homozygous model (AA vs. GG)


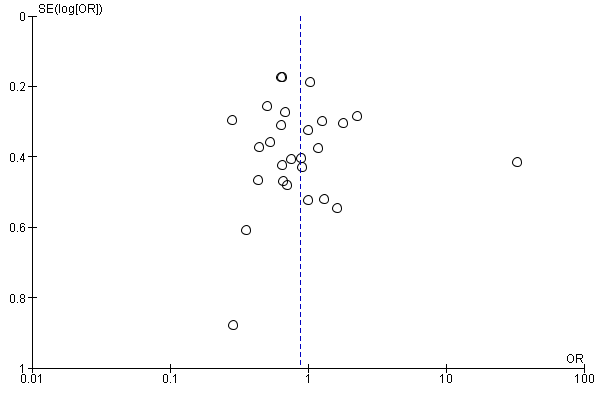


**Figure 8S**: Funnel plot analysis of association between *–308 G/A rs1800629* *polymorphism* and the risk of psoriasis based on heterozygous model (GA vs. GG)


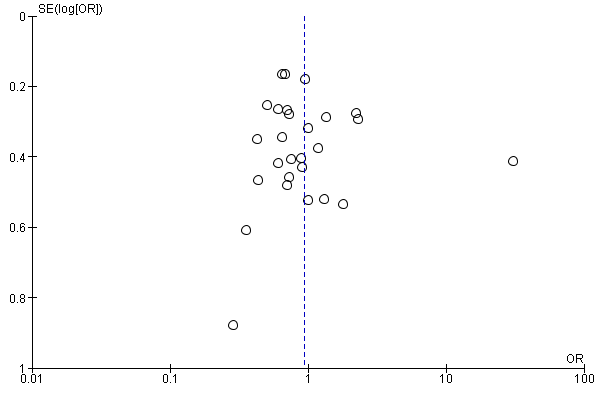


**Figure 9S**: Funnel plot analysis of association between *–308 G/A rs1800629* *polymorphism* and the risk of psoriasis based on dominant model (AA + GA vs. GG)


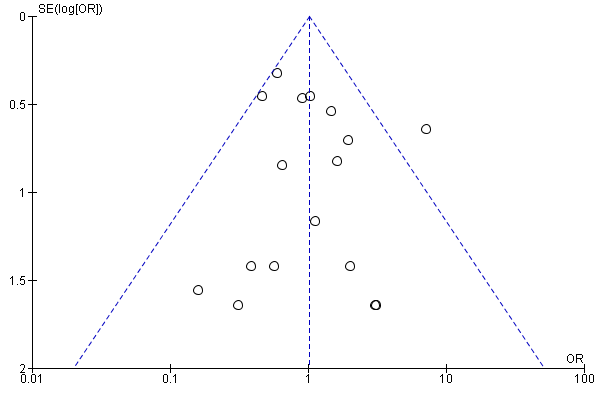


**Figure 10S**: Funnel plot analysis of association between *–308 G/A rs1800629* *polymorphism* and the risk of psoriasis based on recessive model (AA vs. GA + GG)

**
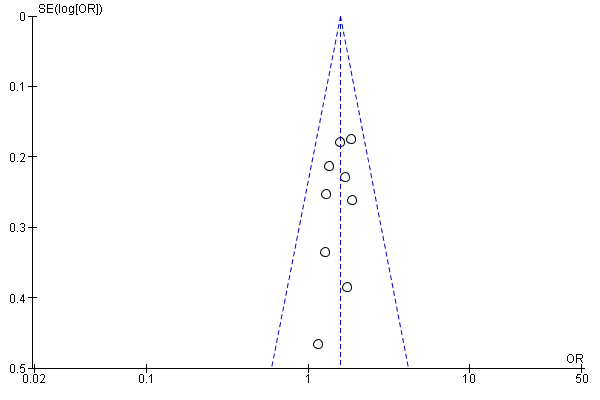
**

**Figure 11S**: Funnel plot analysis of association between *–857 C/T rs1799724 polymorphism* and the risk of psoriasis based on allelic model (T vs. C)


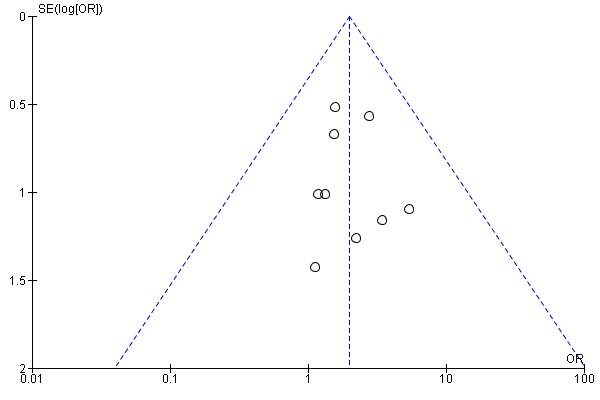


**Figure 12S**: Funnel plot analysis of association between *–857 C/T rs1799724 polymorphism* and the risk of psoriasis based on homozygous model (TT vs. CC)

**
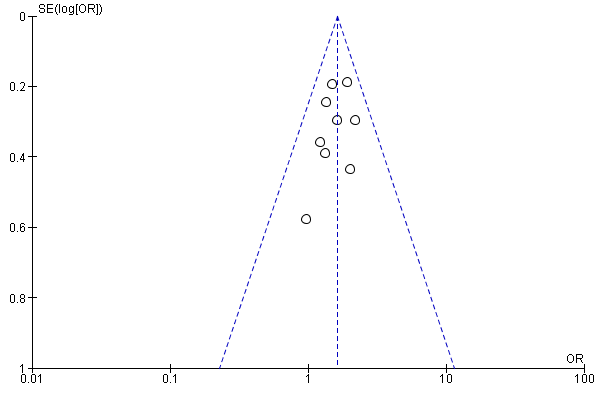
**

**Figure 13S**: Funnel plot analysis of association between *–857 C/T rs1799724 polymorphism* and the risk of psoriasis based on heterozygous model (CT vs. CC)


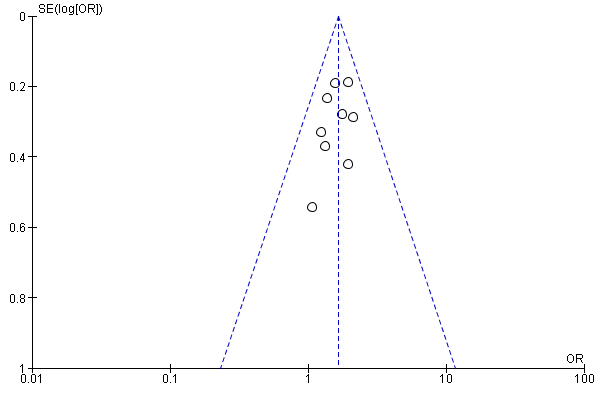


**Figure 14S**: Funnel plot analysis of association between *–857 C/T rs1799724 polymorphism* and the risk of psoriasis based on dominant model (TT + CT vs. CC)


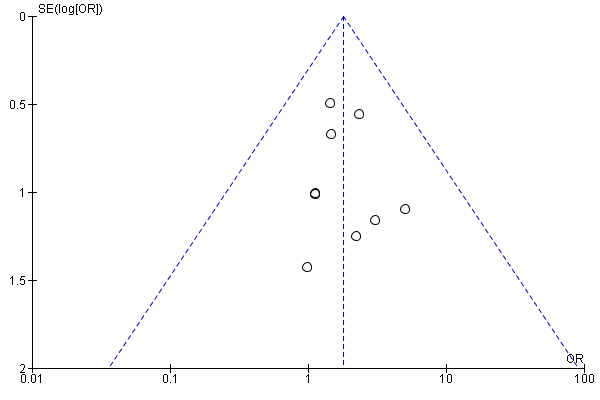


**Figure 15S**: Funnel plot analysis of association between *–857 C/T rs1799724 polymorphism* and the risk of psoriasis based on recessive model (TT vs. CT + CC)

**
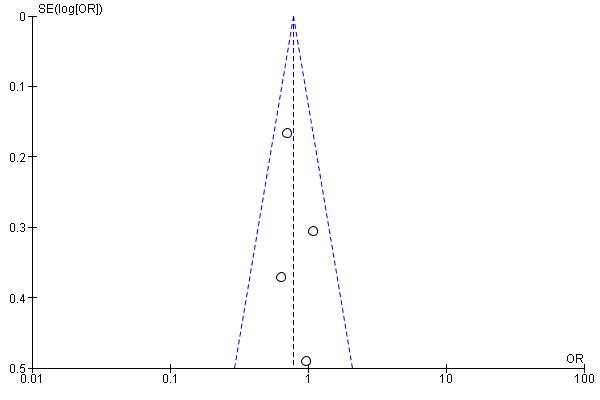
**

**Figure 16S**: Funnel plot analysis of association between *–863 C/A rs1800630 polymorphism* and the risk of psoriasis based on allelic model (A vs. C)


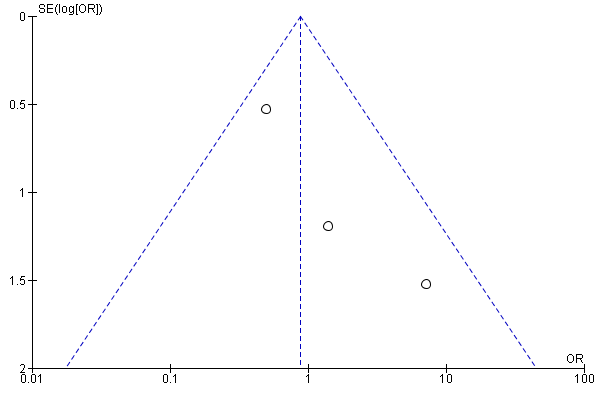


**Figure 17S**: Funnel plot analysis of association between *–863 C/A rs1800630 polymorphism* and the risk of psoriasis based on homozygous model (AA vs. CC)


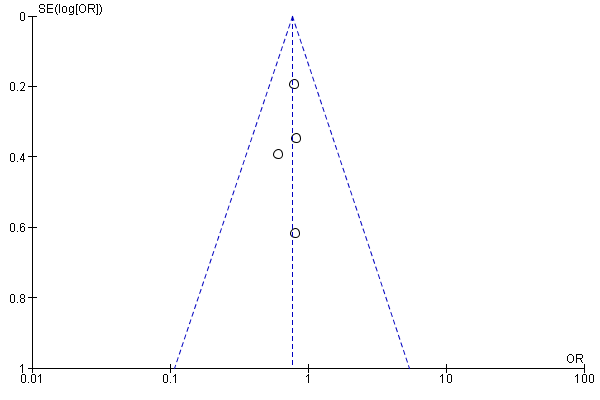


**Figure 18S**: Funnel plot analysis of association between *–863 C/A rs1800630 polymorphism* and the risk of psoriasis based on heterozygous model (CA vs. CC)


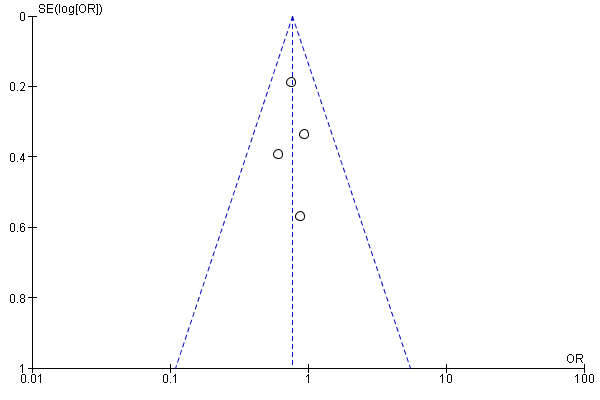


**Figure 19S**: Funnel plot analysis of association between *–863 C/A rs1800630 polymorphism* and the risk of psoriasis based on dominant model (AA + CA cs. CC)


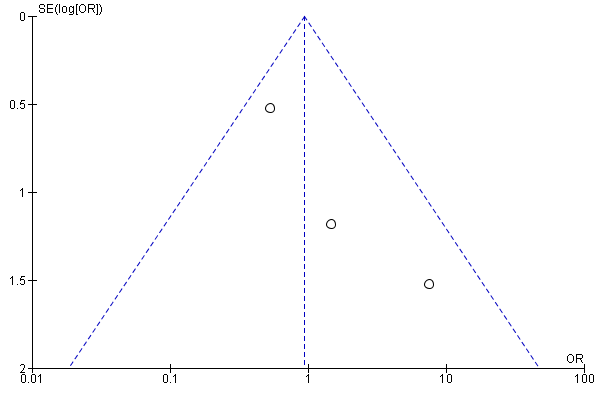


**Figure 20S**: Funnel plot analysis of association between *–863 C/A rs1800630 polymorphism* and the risk of psoriasis based on recessive model (AA vs. CA + CC)


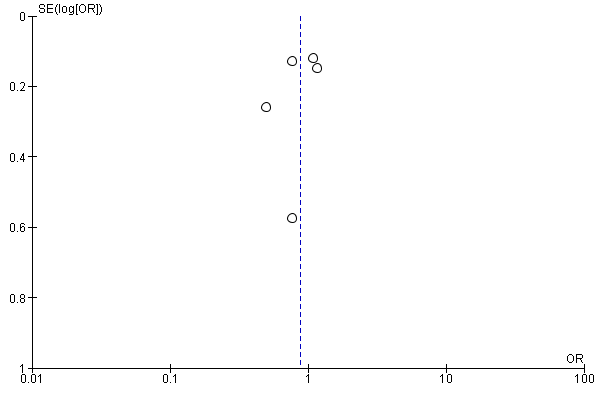


**Figure 21S**: Funnel plot analysis of association between *–1031 T/C rs1799964 polymorphism* and the risk of psoriasis based on allelic model (C vs. T)


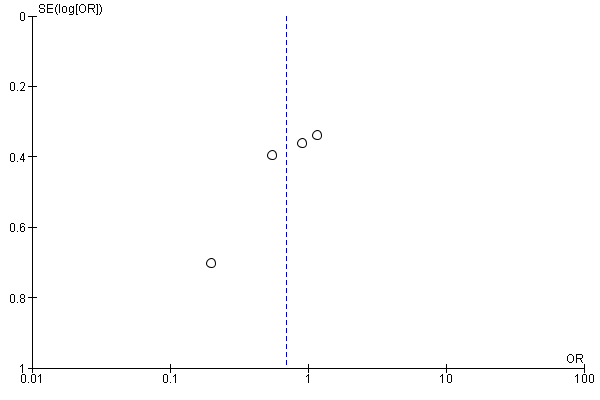


**Figure 22S**: Funnel plot analysis of association between *–1031 T/C rs1799964 polymorphism* and the risk of psoriasis based on homozygous model (CC vs. TT)


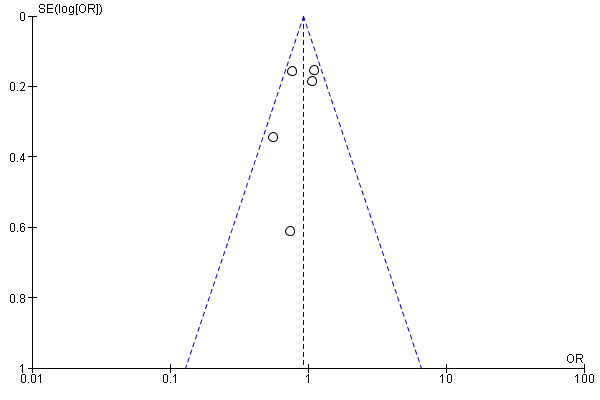


**Figure 23S**: Funnel plot analysis of association between *–1031 T/C rs1799964 polymorphism* and the risk of psoriasis based on heterozygous model (TC vs. TT)


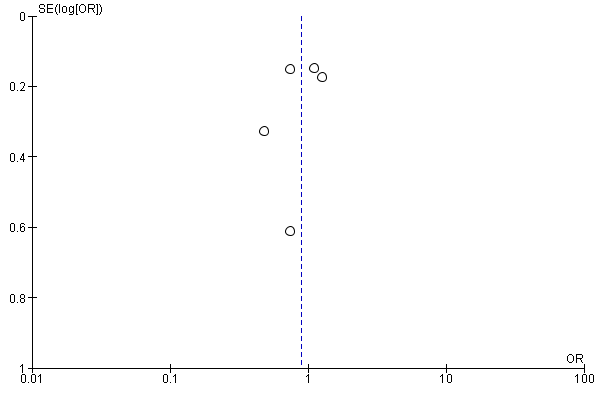


**Figure 24S**: Funnel plot analysis of association between *–1031 T/C rs1799964 polymorphism* and the risk of psoriasis based on dominant model (CC + TC vs. TT)


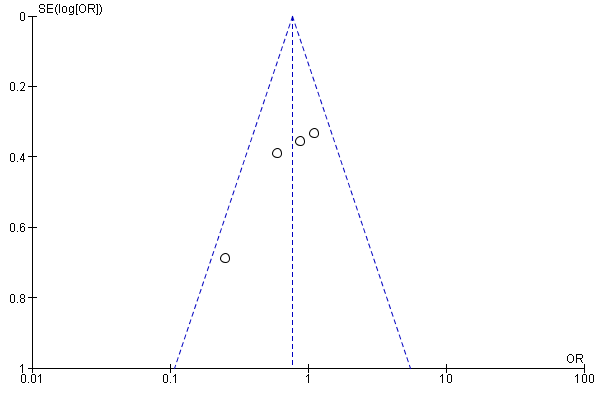


**Figure 25S**: Funnel plot analysis of association between *–1031 T/C rs1799964 polymorphism* and the risk of psoriasis based on recessive model (CC vs. TC + TT)
