# Supplementary material for: Association between tumor necrosis factor-alpha polymorphisms (rs361525, rs1800629, rs1799724, 1800630, and rs1799964) and risk of psoriasis in studies following Hardy-Weinberg equilibrium: A systematic review and meta-analysis
Source: Heliyon. 2023 Jun 22;9(7):e17552. doi: 10.1016/j.heliyon.2023.e17552 (PMC10338315; doi:10.1016/j.heliyon.2023.e17552)
Supplement: Multimedia component 4 [file mmc4.docx]

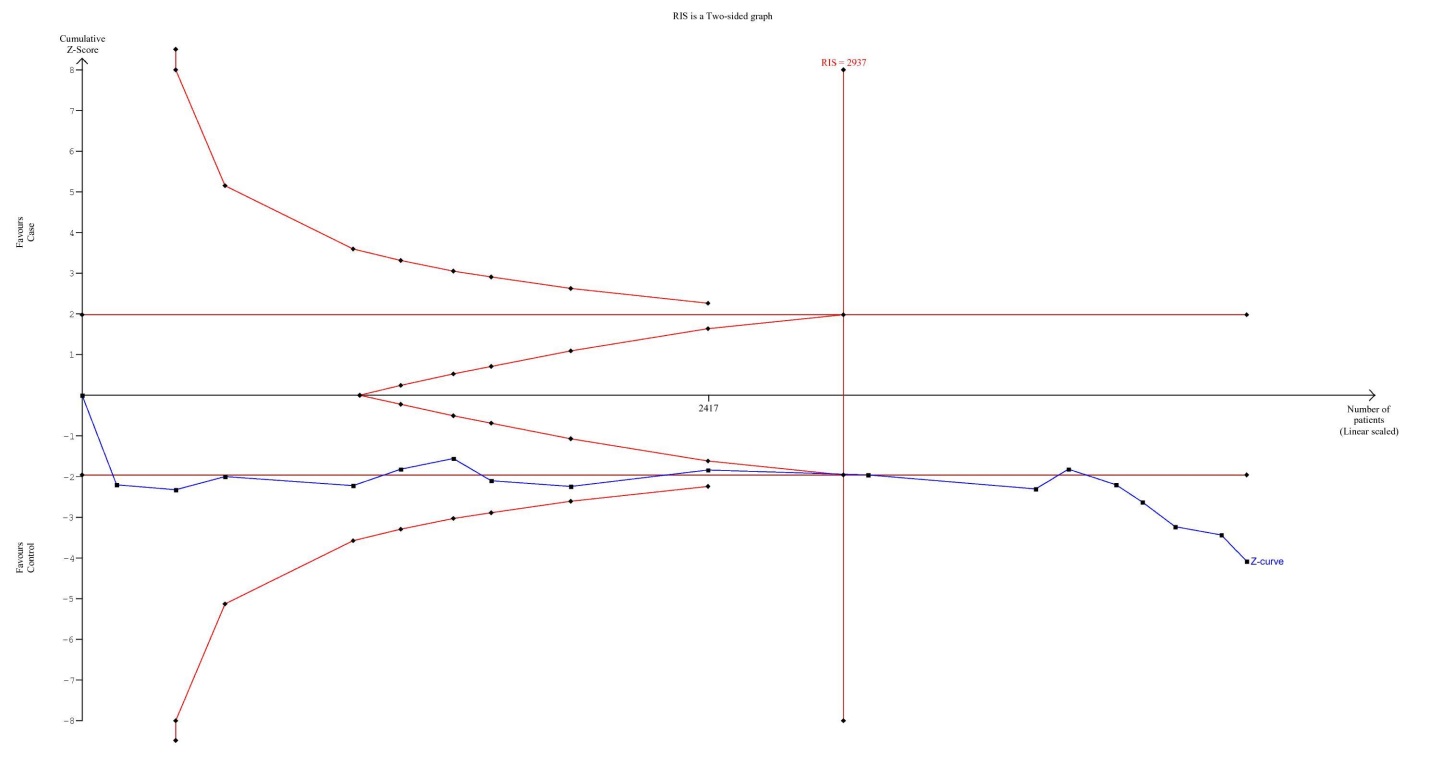


**Figure S1**: Trial sequential analysis for homozygous model of *–238 G/A rs361525* polymorphism (D^2^ = 22%)


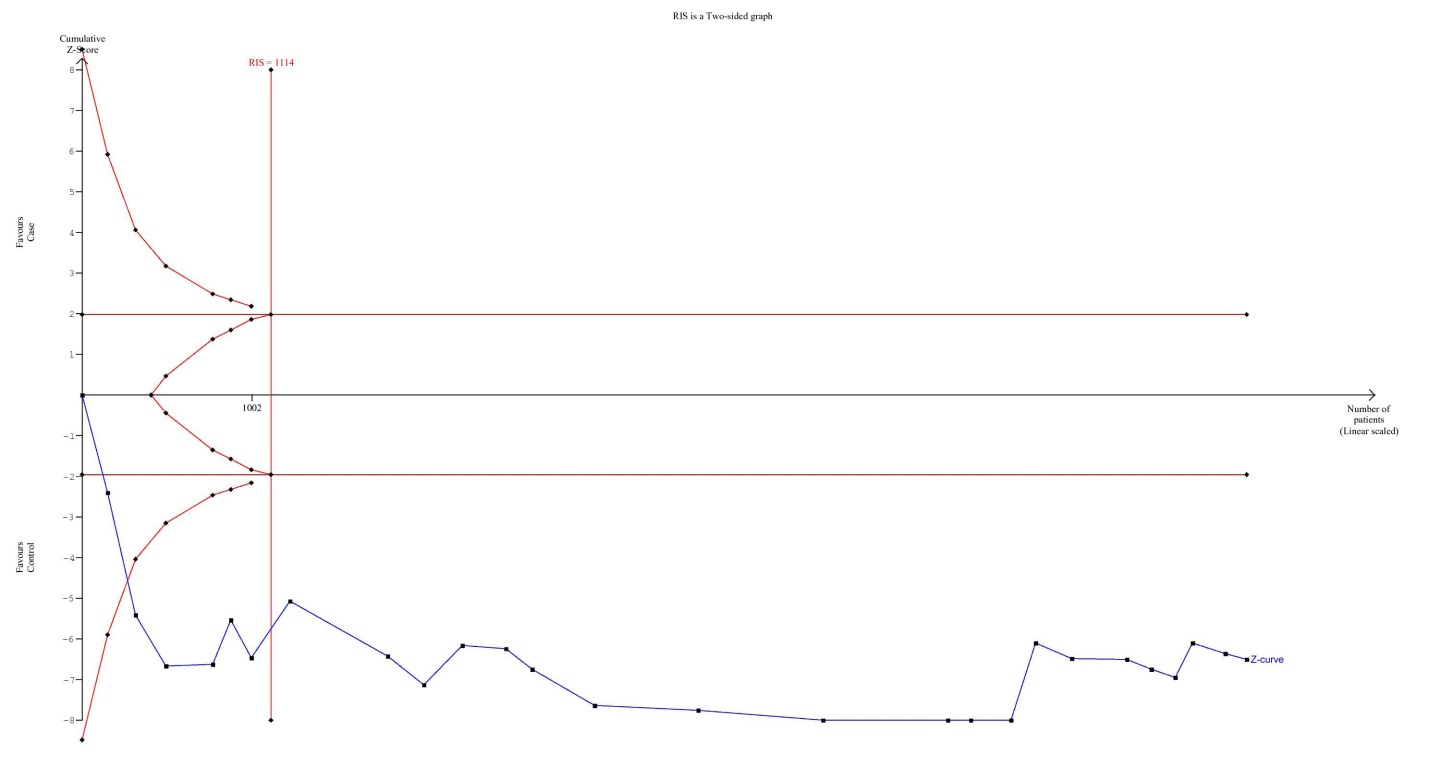


**Figure S2**: Trial sequential analysis for heterozygous model of *–238 G/A rs361525* polymorphism (D^2^ = 68%)


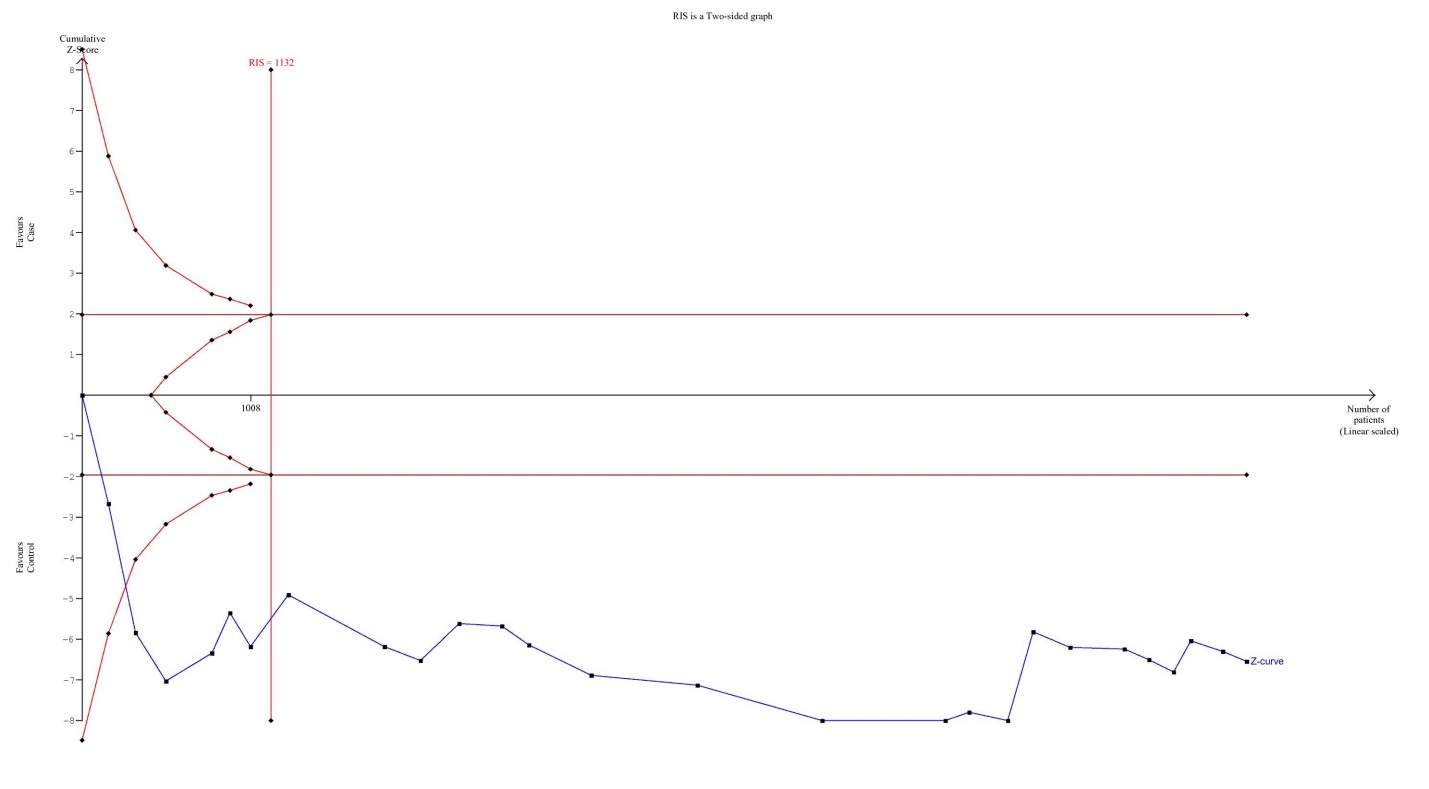


**Figure S3**: Trial sequential analysis for dominant model of *–238 G/A rs361525* polymorphism (D^2^ = 71%)


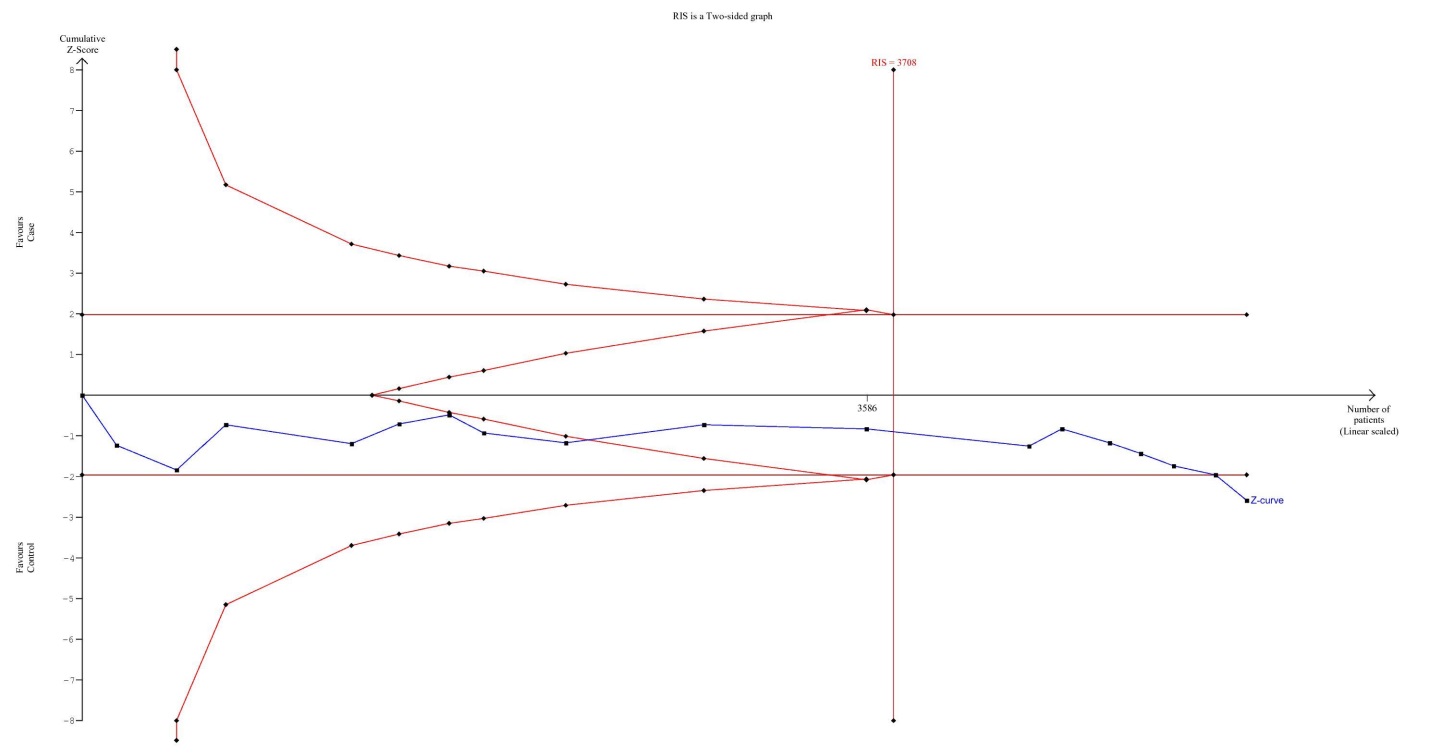


**Figure S4**: Trial sequential analysis for recessive model of *–238 G/A rs361525* polymorphism (D^2^ = 9%)


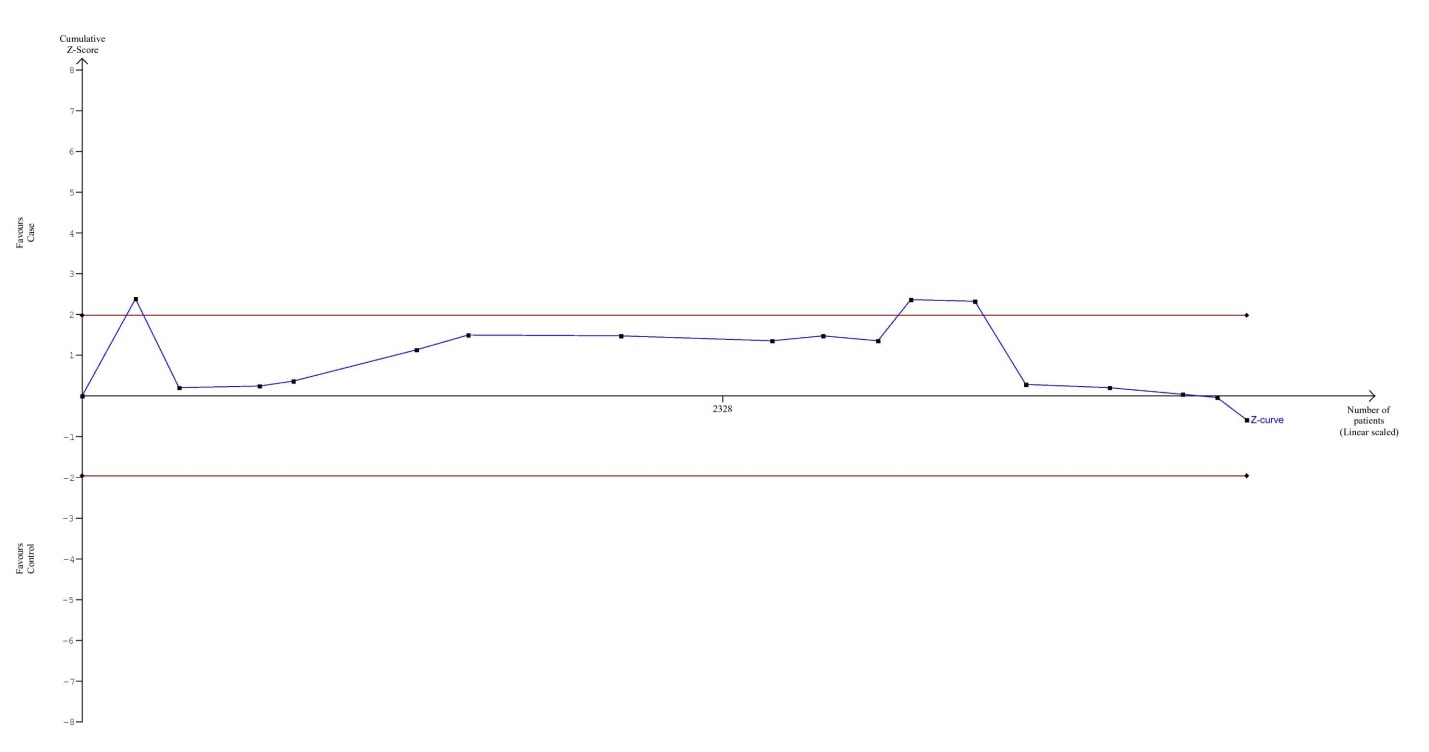


**Figure S5**: Trial sequential analysis for homozygous model of *–238 G/A rs1800629* polymorphism (D^2^ = 72%)


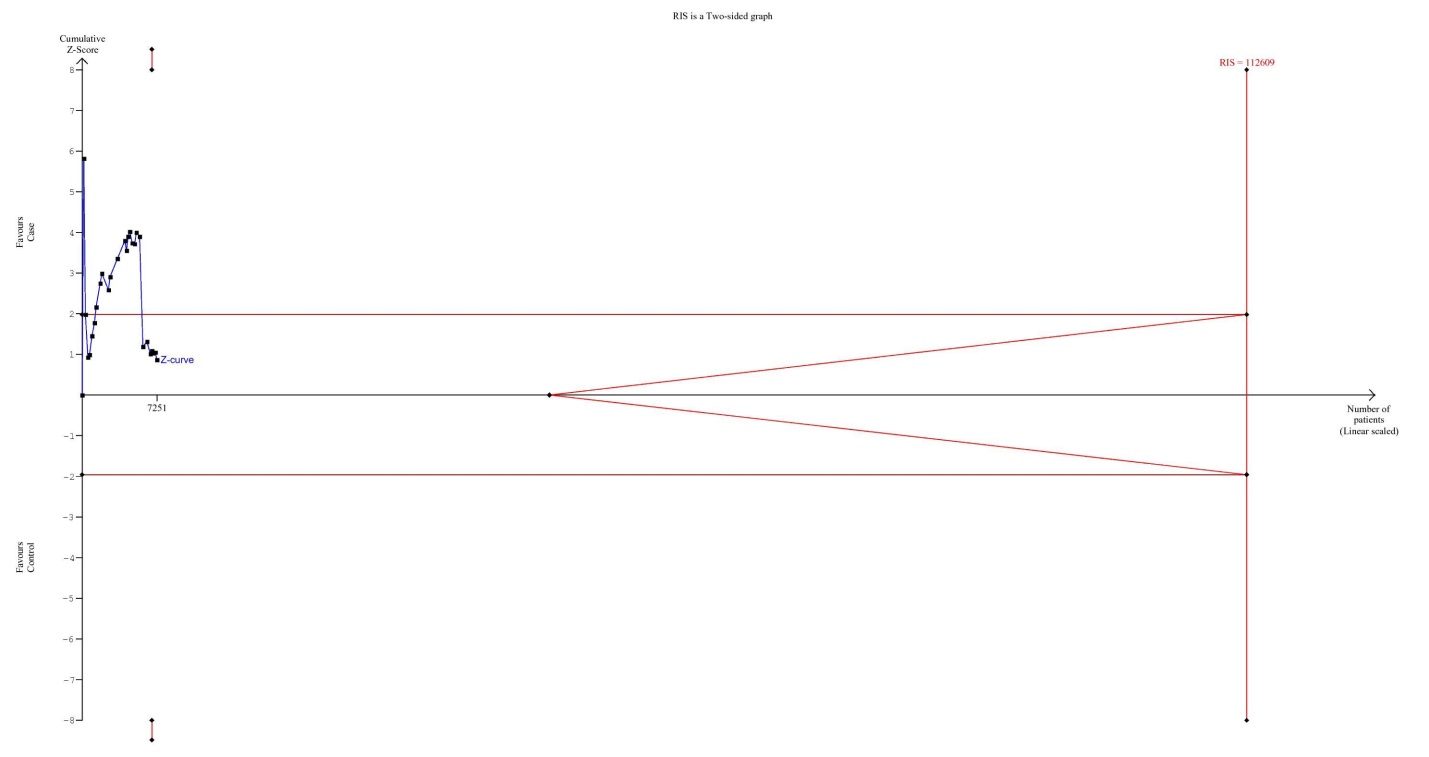


**Figure S6**: Trial sequential analysis for heterozygous model of *–238 G/A rs1800629* polymorphism (D^2^ = 83%)


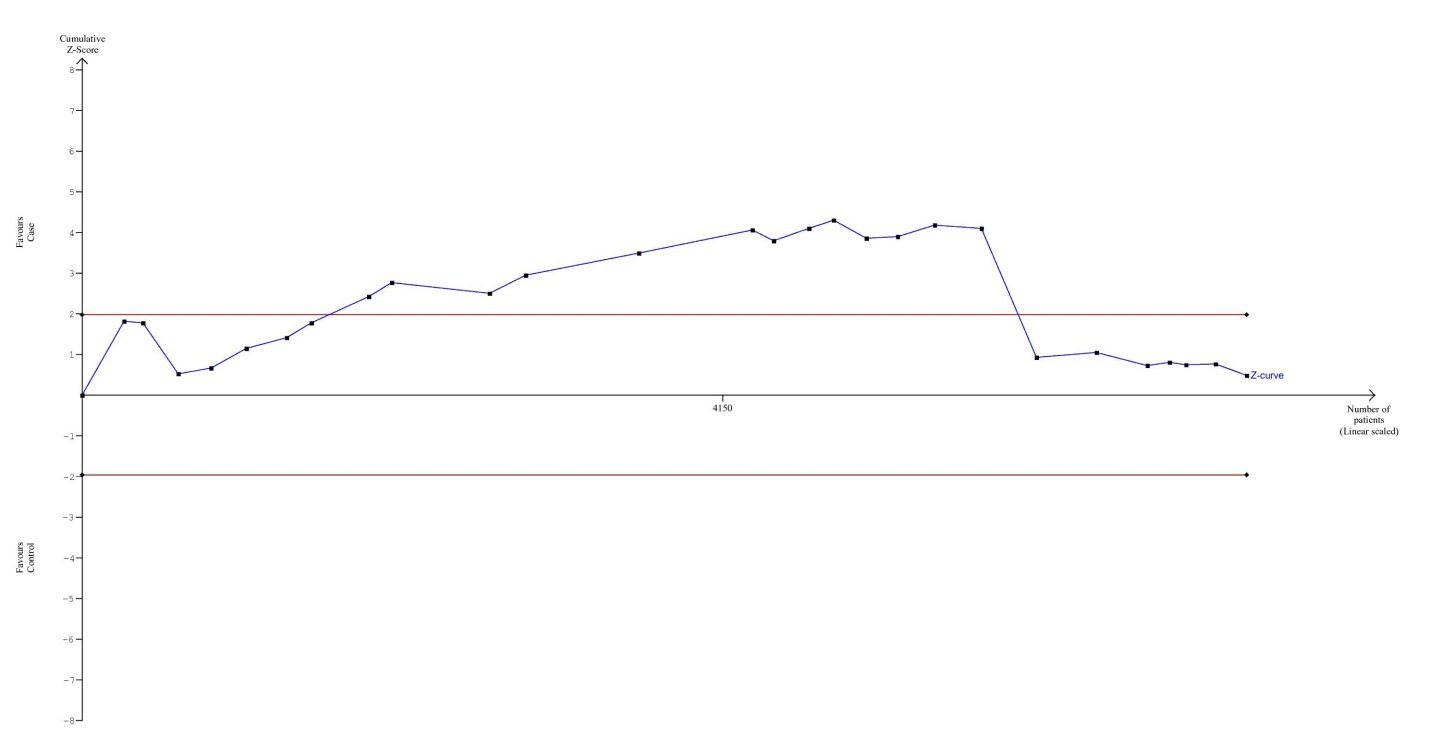


**Figure S7**: Trial sequential analysis for dominant model of *–238 G/A rs1800629* polymorphism (D^2^ = 82%)


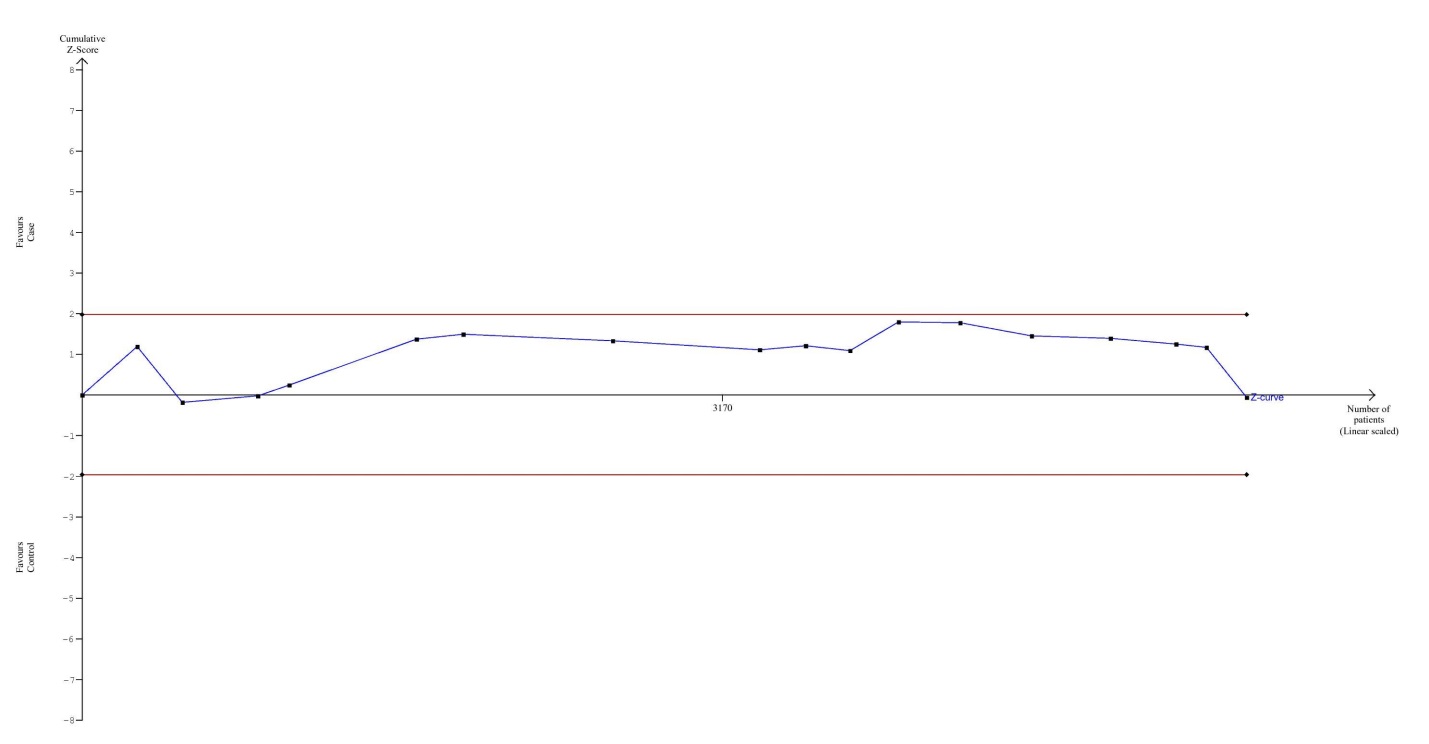


**Figure S8**: Trial sequential analysis for recessive model of *–238 G/A rs1800629* polymorphism (D^2^ = 36%)


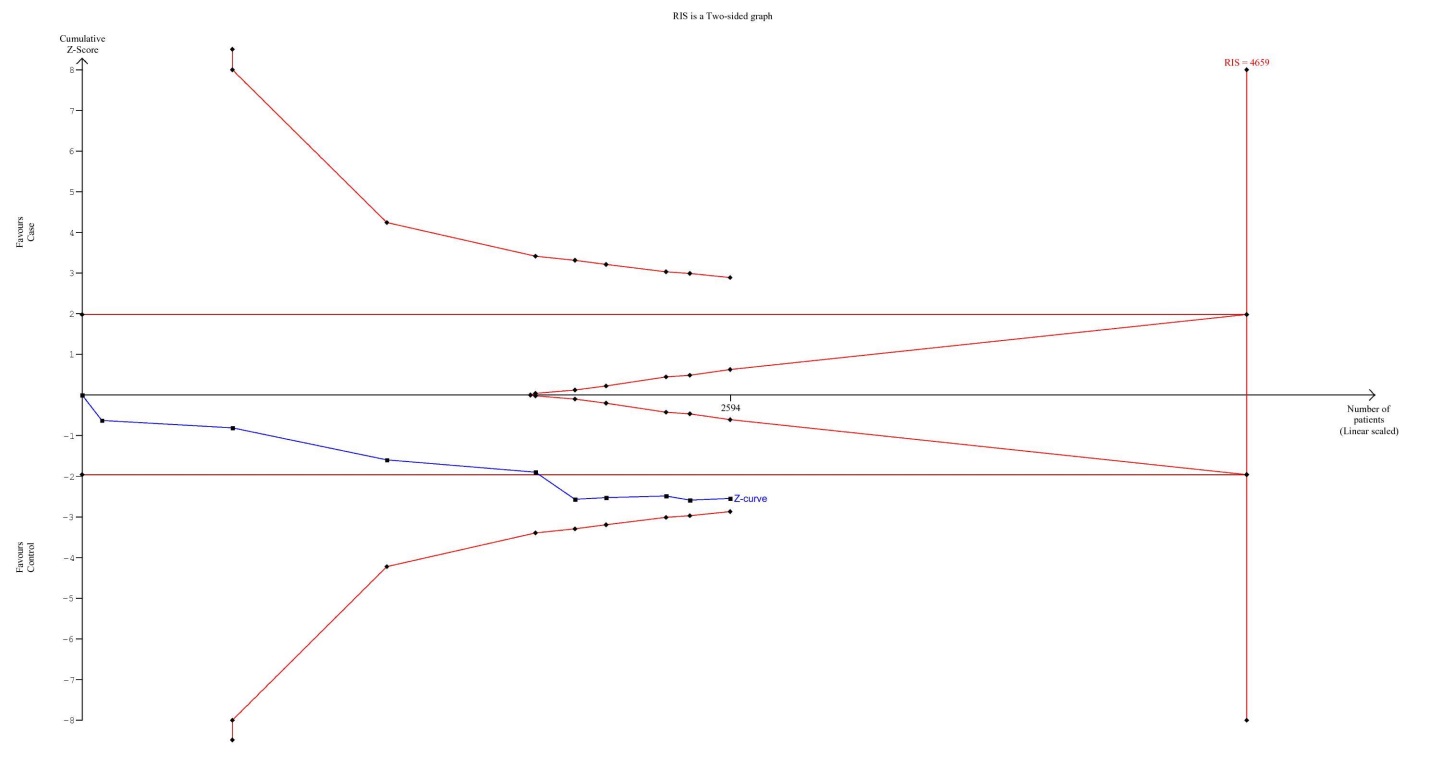


**Figure S9**: Trial sequential analysis for homozygous model of *–238 G/A rs1799724* polymorphism (D^2^ = 0%)


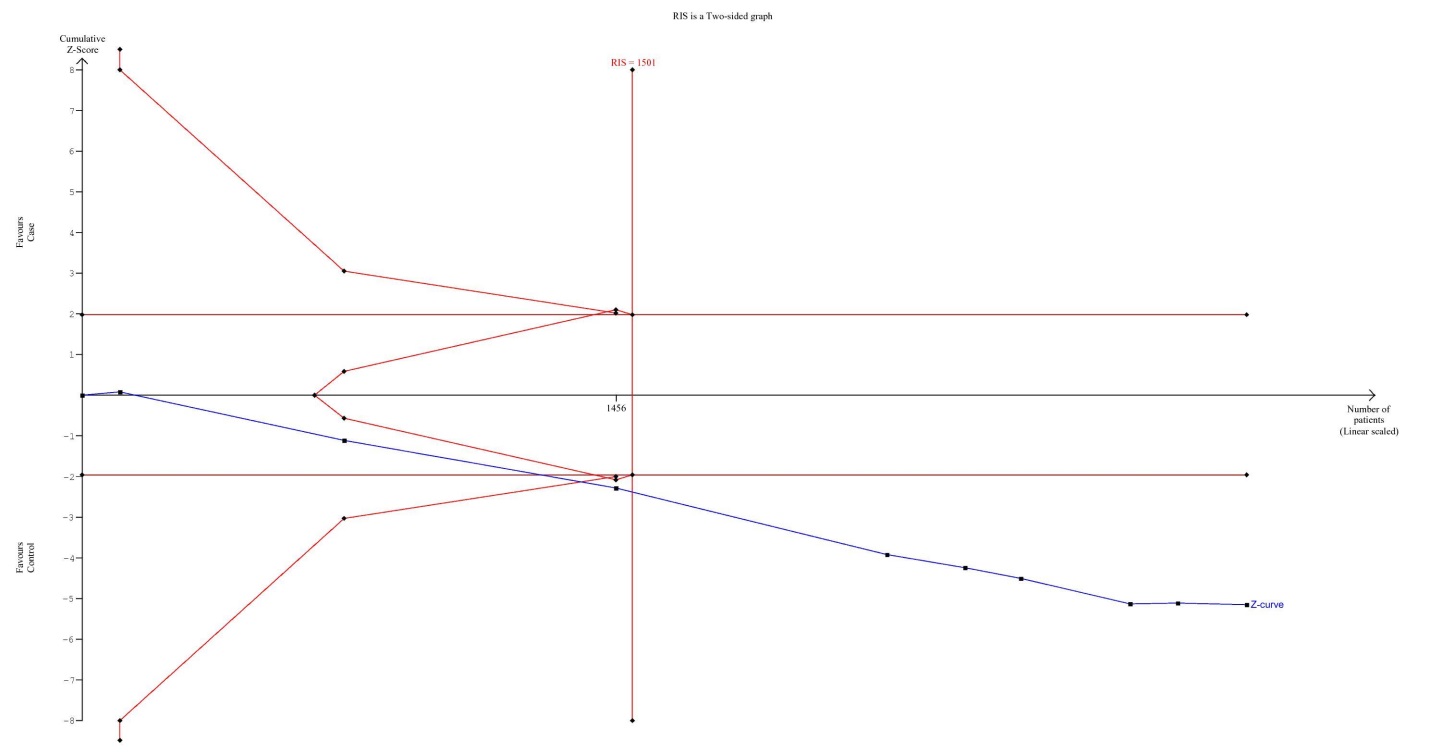


**Figure S10**: Trial sequential analysis for heterozygous model of *–238 G/A rs1799724* polymorphism (D^2^ = 0%)


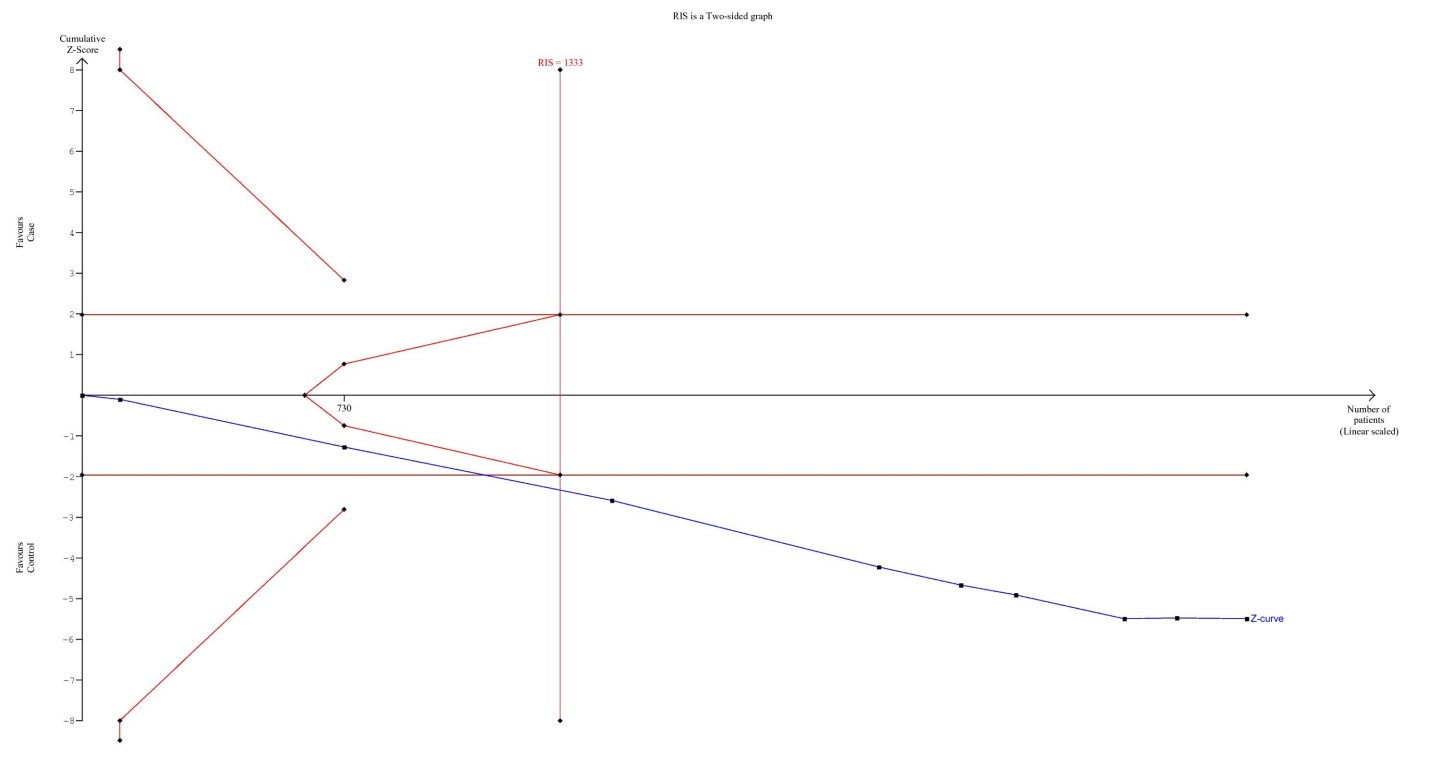


**Figure S11**: Trial sequential analysis for dominant model of *–238 G/A rs1799724* polymorphism (D^2^ = 0%)


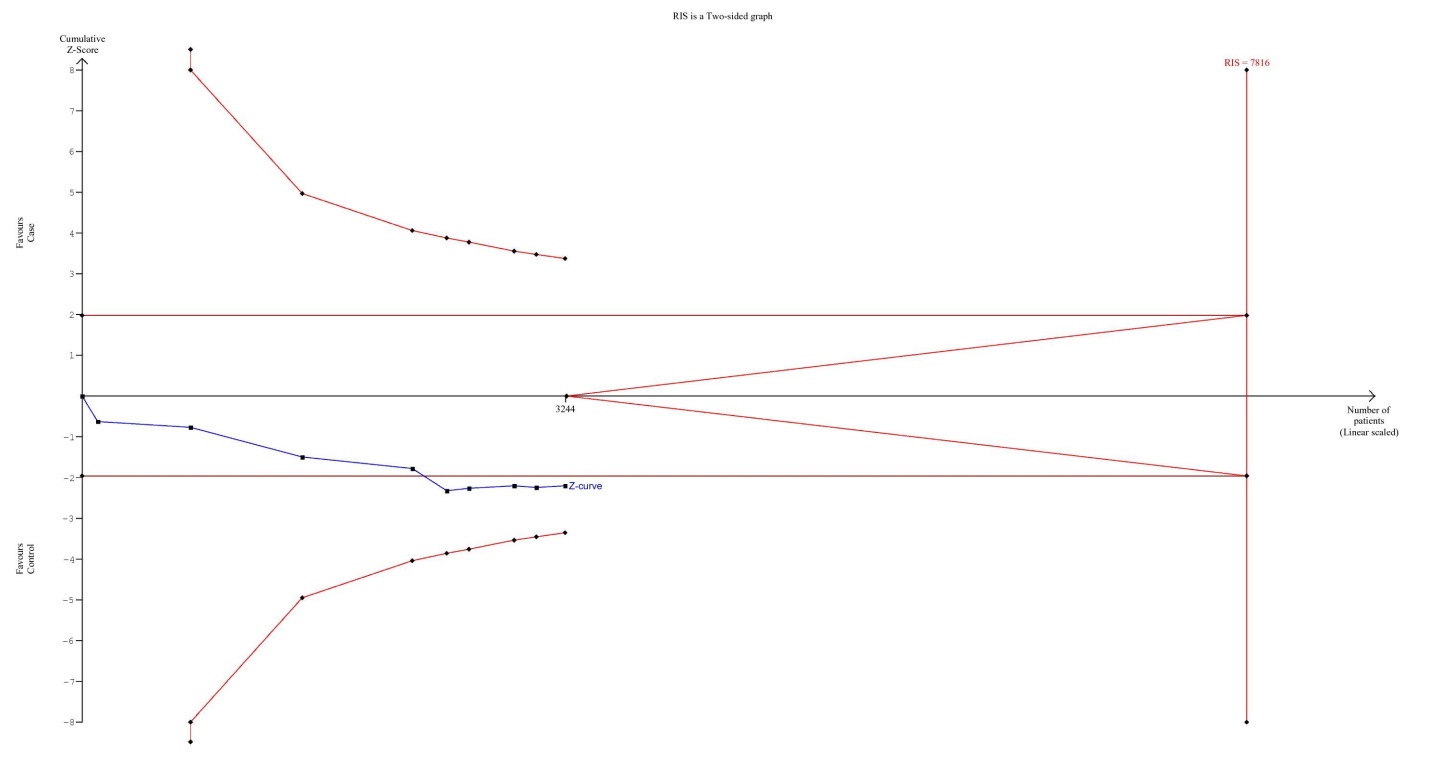


**Figure S12**: Trial sequential analysis for recessive model of *–238 G/A rs1799724* polymorphism (D^2^ = 0%)
